# *Copaifera* spp. oleoresins impair *Toxoplasma gondii* infection in both human trophoblastic cells and human placental explants

**DOI:** 10.1038/s41598-020-72230-0

**Published:** 2020-09-16

**Authors:** Samuel Cota Teixeira, Guilherme de Souza, Bruna Cristina Borges, Thádia Evelyn de Araújo, Alessandra Monteiro Rosini, Fábio Alves Aguila, Sergio Ricardo Ambrósio, Rodrigo Cassio Sola Veneziani, Jairo Kenupp Bastos, Marcelo José Barbosa Silva, Carlos Henrique Gomes Martins, Bellisa de Freitas Barbosa, Eloisa Amália Vieira Ferro

**Affiliations:** 1 Laboratory of Immunophysiology of Reproduction, Institute of Biomedical Science, Federal University of Uberlândia, Campus Umuarama, Av. Para, 1720, Uberlândia, MG 38400239 Brazil; 2 Department of Immunology, Biomedical Sciences Institute, Federal University of Uberlândia, Uberlândia, Brazil; 3 Nucleus of Research in Technological and Exact Sciences, University of Franca, Franca, Brazil; 4 School of Pharmaceutical Sciences of Ribeirao Preto, University of São Paulo, Ribeirão Preto, Brazil; 5 Department of Microbiology, Biomedical Sciences Institute, Federal University of Uberlândia, Uberlândia, Brazil

**Keywords:** Parasitic infection, Pathogens, Parasitic infection, Cell biology

## Abstract

The combination of pyrimethamine and sulfadiazine is the standard care in cases of congenital toxoplasmosis. However, therapy with these drugs is associated with severe and sometimes life-threatening side effects. The investigation of phytotherapeutic alternatives to treat parasitic diseases without acute toxicity is essential for the advancement of current therapeutic practices. The present study investigates the antiparasitic effects of oleoresins from different species of *Copaifera* genus against *T. gondii*. Oleoresins from *C. reticulata*, *C. duckei*, *C. paupera,* and *C. pubiflora* were used to treat human trophoblastic cells (BeWo cells) and human villous explants infected with *T. gondii.* Our results demonstrated that oleoresins were able to reduce *T. gondii* intracellular proliferation, adhesion, and invasion. We observed an irreversible concentration-dependent antiparasitic action in infected BeWo cells, as well as parasite cell cycle arrest in the S/M phase. The oleoresins altered the host cell environment by modulation of ROS, IL-6, and MIF production in BeWo cells. Also, *Copaifera* oleoresins reduced parasite replication and TNF-α release in villous explants. Anti-*T. gondii* effects triggered by the oleoresins are associated with immunomodulation of the host cells, as well as, direct action on parasites.

## Introduction

*Toxoplasma gondii* is an obligate intracellular protozoan parasite belonging to the Apicomplexa phylum^1^. *T. gondii* is the etiologic agent of toxoplasmosis, a zoonotic food-borne infection, which is a significant public health issue worldwide with a broad range of clinical syndromes in humans^2^. Epidemiological surveys show that this intracellular parasite chronically infects 30 to 90% of the global population with substantive differences between countries^3–7^.

Infection with *T. gondii* is usually asymptomatic in healthy individuals, but it can cause severe symptoms in infected children, newborns, and immunocompromised individuals^7^. Infection during or just before pregnancy can result in the vertical transmission of *T. gondii* tachyzoites, which may cross the placenta and invade fetal tissues^8^. The congenital infection may be systemic and can be particularly serious, resulting in miscarriage, stillbirth, fetal death, fetal abnormalities, encephalitis, chorioretinitis, and child disability^8,9^.

The rate of congenital transmission during the first and second trimesters of pregnancy is less than 10 to 30%, respectively, and increases to nearly 90% during of third trimester^10–12^. In contrast, the severity of fetal damage decreases with the gestational progression^13,14^. The placental barrier is more efficient in inhibiting vertical transmission of *T. gondii* tachyzoites at the beginning of gestation but becomes more susceptible at the end of pregnancy^15^.

Pregnant women infected by *T. gondii* require early diagnosis, and anti-parasitic treatment in order to improve both mother and child health^12^. The current literature shows that early treatment of the infected mother could prevent or reduce vertical transmission and, consequently, the fetal damage^12,16–18^. When maternal infection by *T. gondii* is detected, and there is no evidence of fetal infection, the common therapeutic practice indicates the use of spiramycin, a macrolide antibiotic that prevents the congenital transmission^8,19,20^. However, this macrolide does not cross the placenta and is not suitable for treatment when a fetal infection is confirmed^21^.

In cases of congenital toxoplasmosis, a combination of pyrimethamine and sulfadiazine is the first choice for treatment. When combined, the drugs act in synergism to inhibit crucial enzymes involved in the biosynthesis of *T. gondii* pyrimidines, which are essentials for both parasite survival and replication^22–24^. Despite the importance of these drugs to control infection by *T. gondii*, their use is linked to serious toxicity, such as gradual dose-related bone marrow depression, gastrointestinal disorders, and teratogenic effects on the fetus during the first trimester of pregnancy^3,25–27^.

During the last decade, our research group has been making efforts to find more effective and less toxic therapeutic alternatives for the treatment of congenital toxoplasmosis. In this context, our group showed that azithromycin treatment reduced the vertical transmission of *T. gondii* tachyzoites in pregnant *Calomys callosus* rodents and was able to control parasite infection in human trophoblastic cells (BeWo cells)^28,29^. Moreover, we demonstrated that azithromycin treatment promoted inhibition of proliferation of *T. gondii* Brazilian strains in human villous explants from the third trimester of pregnancy^30,31^. Also, our work with other compounds showed that both enrofloxacin and toltrazuril impairs *T. gondii* infection in vitro, ex vivo*,* and in vivo experimental models^32,33^.

In summary, conventional therapy for congenital toxoplasmosis suppresses the active infection; however, it does not cure the latent infection^34,35^. Moreover, treatment options include the use of drugs, which can cause serious side effects in both mother and child, leading to discontinuation of therapy in up to 40% of patients^34,35^. Thus, current treatment for congenital toxoplasmosis is still limited, affecting mortality and quality of life on pregnancy and neonatal health^7^.

In this scenario, it is relevant to consider plant-derived compounds as the source of new bioactive substances for the treatment of congenital toxoplasmosis^36^. The search for alternative therapeutic tools gathered great interest in the past few decades, where plants with medicinal properties are systematically screened for their potential to treat parasitic diseases^37–41^. Several studies have evaluated the anti-*Toxoplasma* effects of many plant-based products, and promising results have been published^39–48^.

The *Copaifera* genus belongs to the Fabaceae family (Leguminosae) and is present throughout the American and African continents. Their oleoresins are obtained by tapping the trunk of trees and have been extensively studied because of its medicinal properties^49^. These oleoresins exhibit remarkable biological properties such as antimicrobial, anti-inflammatory, and antiparasitic activity^49–53^. However, no current studies investigated the impact of oleoresins from *Copaifera* genus in *T. gondii* infection. The present work investigates the antiparasitic effects of oleoresins from different species of *Copaifera* genus against *T. gondii*. We used two distinct experimental models to assess the effect of *Copaifera* oleoresins: an in vitro model using human trophoblastic cells (BeWo cells) as host cells and an ex vivo model using human villous explants from the third trimester of pregnancy.

## Results

### Oleoresin treatments altered viability in BeWo cells at higher concentrations

**Figure 1:**
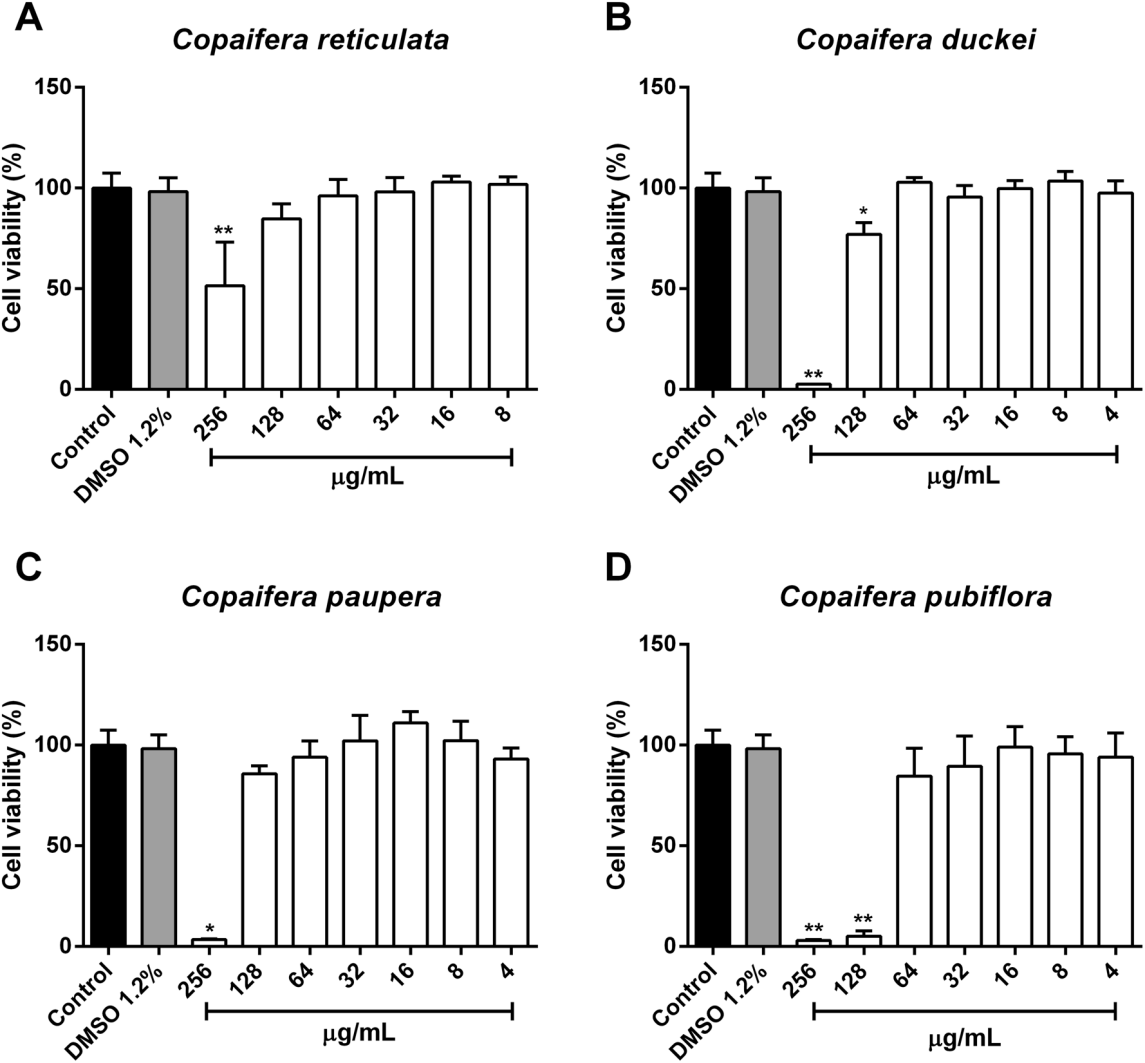
Host cell viability by MTT assay. BeWo cells were treated for 24 h in twofold serial dilutions (ranging from 256 to 4 µg/mL) with oleoresins from (**A**) *Copaifera reticulata*, (**B**) *Copaifera duckei*, (**C**) *Copaifera paupera* and (**D**) *Copaifera pubiflora.* BeWo cells were treated with culture medium alone (control group) and DMSO 1.2% (vehicle of oleoresins in the concentration of 256 µg/mL). Cell viability was expressed in percentages (cell viability %), with the absorbance of cells incubated only with culture medium considered to be 100% viability. Data are expressed as means ± standard deviation from experiments performed in eight replicates. Significant differences detected by the Kruskal–Wallis test and Dunn’s multiple comparison post-test are labeled (statistically significant when *P* < 0.05).

Evaluation of the oleoresin impact in cell viability, human trophoblastic cells (BeWo lineage) were treated with four oleoresins extracted from different species from *Copaifera* spp., as follows: *Copaifera reticulata*, *Copaifera duckei*, *Copaifera paupera* and *Copaifera pubiflora* (Fig. 1). BeWo cells exposed to oleoresins in different concentration only showed loss of viability at 24 h after treatment, and only at high doses. The minimal dose for toxic effect, as measured by loss of viability, was 256 µg/mL for oleoresins of *C. reticulata* (^**^P = 0.0063), *C. paupera* (^*^P = 0.0138) (Fig. 1A,C), and 128 µg/mL for *C. duckei* (^*^P = 0.032), and *C. pubiflora* (^**^P = 0.0015) (Fig. 1B,D). All treated experiments were compared to cells incubated only with culture medium (control group). As vehicle control, BeWo cells treated with DMSO 1.2% did not present loss of cell viability.

### *Copaifera* oleoresins significantly reduced *T. gondii* intracellular proliferation in BeWo cells

**Figure 2:**
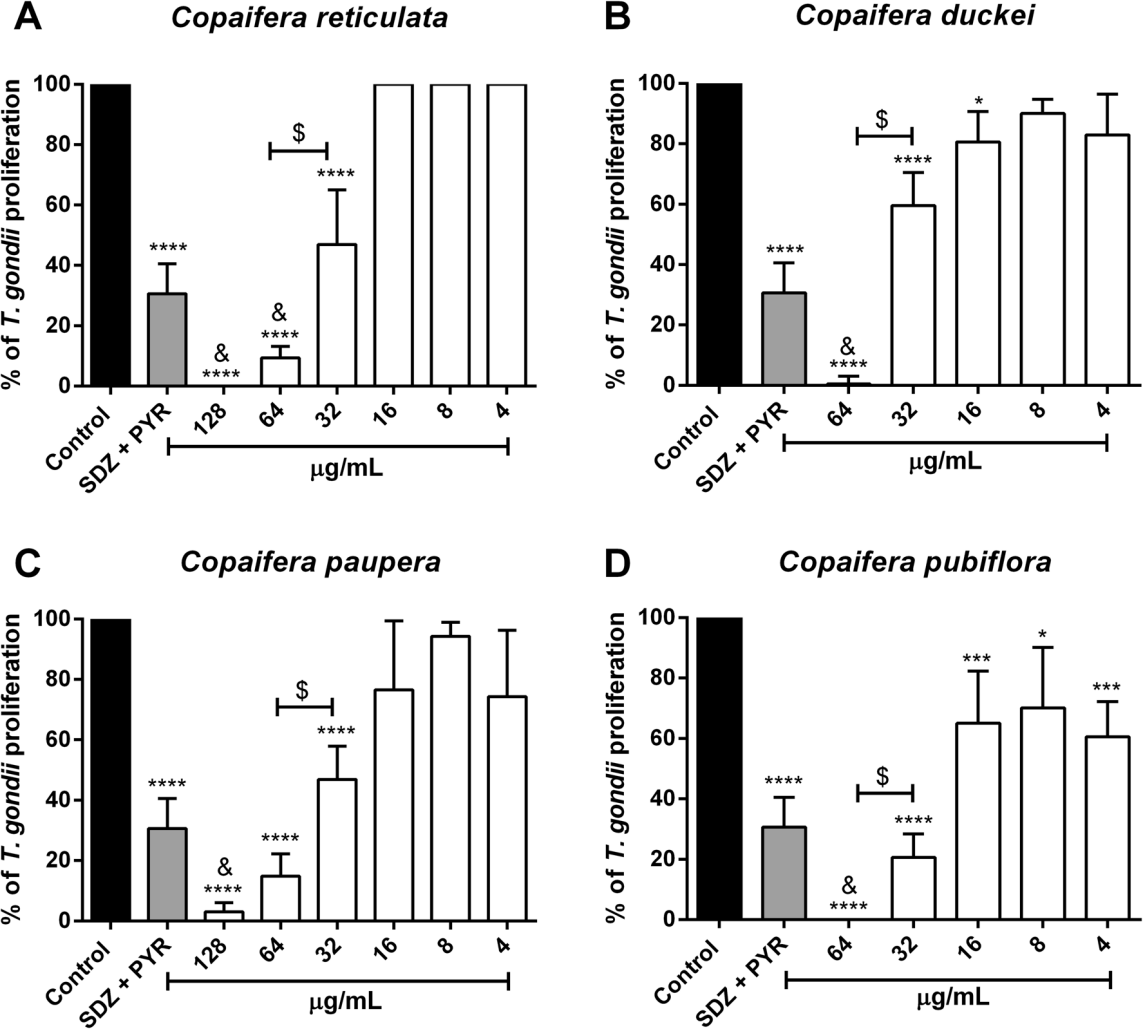
β-galactosidase activity-based screen. *T. gondii*-infected BeWo cells were treated for 24 h with non-toxic concentrations in twofold serial dilutions of oleoresins from (**A**) *Copaifera reticulata* (ranging from 128 to 4 µg/mL), (**B**) *Copaifera duckei* (ranging from 64 to 4 µg/mL), (**C**) *Copaifera paupera* (ranging from 128 to 4 µg/mL) and (**D**) *Copaifera pubiflora* (ranging from 64 to 4 µg/mL). We treated BeWo cells with culture medium only (control group—considered as 100% parasite proliferation), or a combination of 200 µg/mL of sulfadiazine and 8 µg/mL of pyrimethamine (SDZ + PYR). *T. gondii* intracellular proliferation was analyzed using a colorimetric β-galactosidase assay and expressed in percentage change compared to control (% of *T. gondii* proliferation). Our results are shown as means ± standard deviation from experiments performed in eight replicates. ^*^Comparison between infected/untreated cells and infected/treated cells; ^&^Comparison to SDZ + PYR-infected/treated cells; ^$^Comparison between both treatment concentrations of each *Copaifera* oleoresin. We used One-Way ANOVA and Turkey’s multiple comparisons post-test to evaluate significant differences (*P* < 0.05).

In our host cell viability assay, we established concentrations of each oleoresin that did not alter the viability of BeWo cells for further set up of experiments. We performed the assays using twofold serial dilutions of oleoresins from *C. reticulata*, *C. paupera* (ranging from 128 to 4 µg/mL), and *C. duckei*, *C. pubiflora* (ranging from 64 to 4 µg/mL). Quantification of *T. gondii* growth inhibition was performed by incubating these selected oleoresin concentrations with infected BeWo cells for 24 h. Parasite growth (% of *T. gondii* proliferation) was quantified by measuring the β-galactosidase activity of viable parasites. All tested oleoresins inhibited parasite proliferation, as follow: *C. reticulata* (128, 64 and 32 µg/mL; ^****^P < 0.0001) (Fig. 2A), *C. duckei* (64 and 32 µg/mL; ^****^P < 0.0001|16 µg/mL; ^*^P = 0.0372) (Fig. 2B), *C. paupera* (128, 64 and 32 µg/mL; ^****^P < 0.0001) (Fig. 2C), *C. pubiflora* (64 and 32 µg/mL; ^****^P < 0.0001|16, 8 and 4 µg/mL; ^***^P = 0.0009, ^*^P = 0,0,361 and ^***^P = 0.0001, respectively) (Fig. 2D), when compared to untreated and infected BeWo cells (control group).

Doses of 128, 64, and 32 µg/mL of all *Copaifera* oleoresins tested displayed a significant reduction of *T. gondii* intracellular proliferation while staying safely below the toxic dose for the cells. The concentration of 64 µg/mL was more efficient to inhibit parasite replication (~ 85–100%) compared to the concentration of 32 µg/mL (~ 40–80%), as follow: *C. reticulata*, *C. duckei*, *C. paupera* (^$^*P* < 0.0001) and *C. pubiflora* (^$^*P* = 0.0060) (Fig. 2). The combination of sulfadiazine and pyrimethamine (SDZ + PYR) (200 + 8 μg/mL, respectively) was also able to reduce parasite replication (~ 70%) in comparison with untreated and infected cells (^****^P < 0.0001) (Fig. 2). In comparison with classical drugs commonly used to treat congenital toxoplasmosis (SDZ + PYR), treatment with 128 and 64 µg/mL of oleoresins from *C. reticulata* (^&^*P* < 0.0001 and ^&^*P* = 0.0007, respectively), and 64 µg/mL *C. duckei* (^&^*P* < 0.0001) were significantly more effective to control parasite growth (~ 90–100%) (Fig. 2A,B). In addition, both 128 µg/mL *C. paupera* (^&^*P* < 0.0007) and 64 µg/mL *C. pubiflora* (^&^*P* < 0.0001) oleoresins were also significantly more effective to inhibit parasite proliferation (~ 95–100%) in relation to SDZ + PYR treatment (~ 70%) (Fig. 2C,D).

### Pre-treatment of *T. gondii* tachyzoites with oleoresins impairs adhesion, invasion and subsequent proliferation

**Figure 3:**
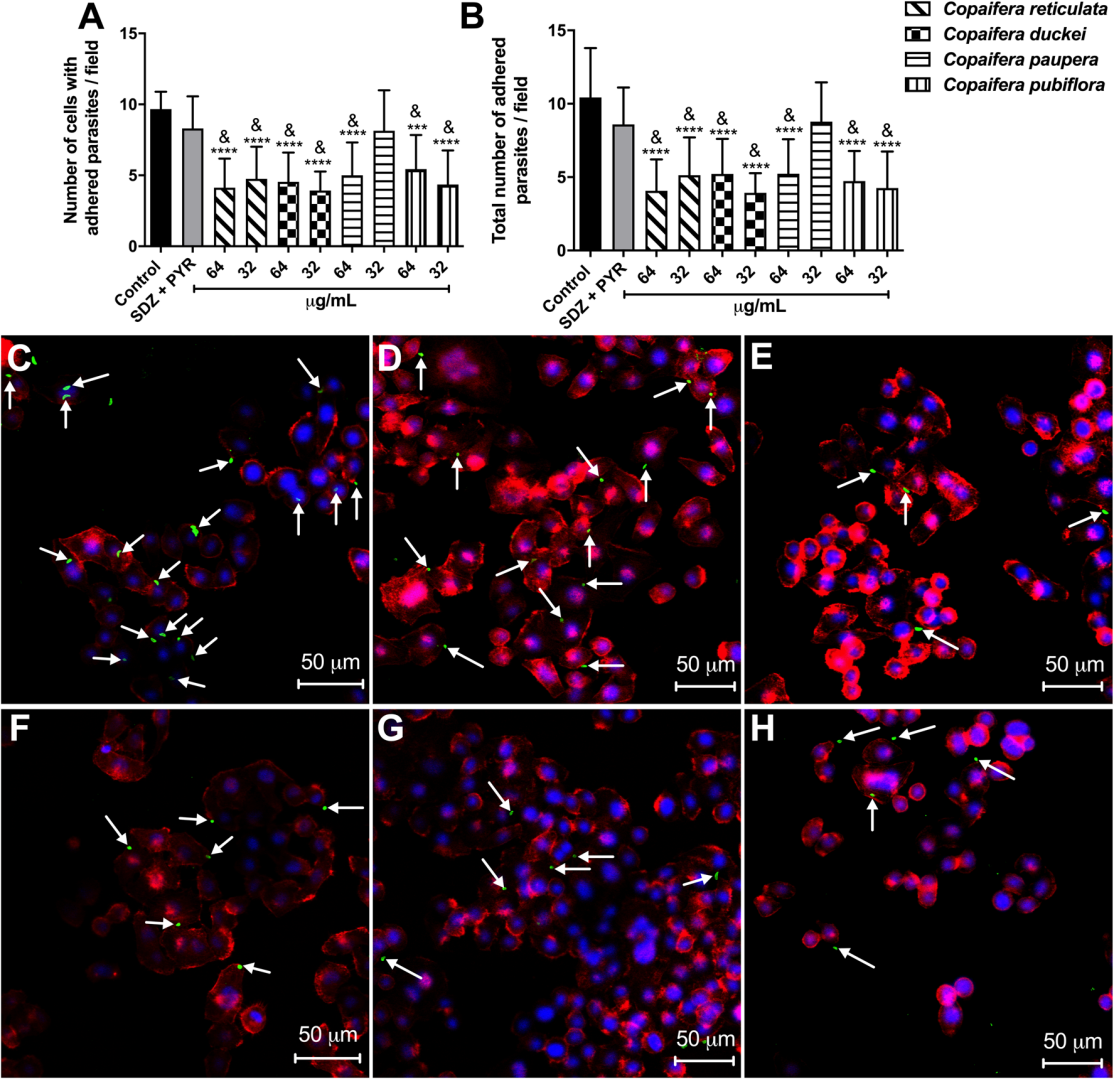
Adhesion assay of *T. gondii* tachyzoites to BeWo cells. *T. gondii* tachyzoites were pre-incubated for one h with 64 and 32 µg/mL of oleoresins from *C. reticulata*, *C. duckei*, *C.* paupera, *C. pubiflora*, SDZ + PYR (200 + 8 μg/mL) and culture medium only (control group) and then allowed to interact for three h with previously fixed BeWo cells. (**A**) The number of cells with adhered parasites and (**B**) the total number of adhered parasites to BeWo cells in a total of approximately 20 fields examined randomly. Representative images highlighting the tachyzoites-host cell interaction are presented according to the conditions analyzed: (**C**) control group (untreated parasites), (**D**) SDZ + PYR, (**E**) *C. reticulata*, (**F**) *C. duckei*, (**G**) *C. paupera* and (**H**) *C. pubiflora*. Representative images of oleoresin treatments showed only the concentration of 64 µg/mL. Data are expressed as means ± standard deviation. The experiment was performed with four replicates. ^*^Comparison between untreated parasites and treated parasites; ^&^Comparison to SDZ + PYR-treated parasites; ^$^Comparison between either treatment concentrations of each *Copaifera* oleoresin. Significant differences were determined by using one-way ANOVA and Tukey’s multiple comparisons post-test. Differences were considered significant when *P* < 0.05. White arrows indicate tachyzoites adhered to BeWo cells. The cell nucleus is labeled with DAPI (blue). *T. gondii* tachyzoites labeled with Alexa Fluor 488-conjugated anti-mouse IgG are shown green. F-actin labeled with Phalloidin-TRITC is shown red. Scale bar: 50 µm.

The success in the establishment of infection by *T. gondii* requires the ability of the parasites to attach and invade host cells^54,55^. To evaluate whether the oleoresin treatments target the host cell or the parasite, we performed a variety of assays. In the first set of experiments, we assessed whether oleoresin treatments would affect the parasite adhesion to host cells. *T. gondii* tachyzoites were pre-incubated with different oleoresins and then incubated with previously fixed BeWo cells. We observed that the pre-treatment of parasites with 64 and 32 µg/mL oleoresins from *C. reticulata* (^****^P < 0.0001), *C. duckei* (^****^P < 0.0001), *C. paupera* (64 µg/mL; ^****^P < 0.0001) and *C. pubiflora* (^***^P < 0.0006 and ^****^P < 0.0001, respectively) reduced their cellular adherent capacity compared to untreated parasites (control group) (Fig. 3A). Interestingly, the pre-treatment of parasites with 64 and 32 µg/mL of oleoresins significantly reduced the number of BeWo cells with parasites attached to them, in comparison to parasites treated with SDZ + PYR. Data from *C. reticulata* (^&^*P* = 0.0003 and ^&^*P* = 0.0039, respectively), *C. duckei* (^&^*P* = 0.0031 and ^&^*P* = 0.0001, respectively), *C. paupera* (64 µg/mL; ^&^*P* < 0.0143) and *C. pubiflora* (^&^*P* < 0.0267 and ^&^*P* < 0.0010, respectively) are shown in Fig. 3A. Also, the pre-treatment of parasites with 64 and 32 µg/mL oleoresins from *C. reticulata* (^****^P < 0.0001), *C. duckei* (^****^P < 0.0001), *C. paupera* (64 µg/mL; ^****^P < 0.0001) and *C. pubiflora* (^****^P < 0.0001) promoted a reduction in the number of adhered parasites to BeWo cells (Fig. 3B). The oleoresin treatments with 64 and 32 µg/mL showed a greater reduction in the number of adhered parasites when compared to the standard treatment with SDZ + PYR. Data from *C. reticulata* (^&^*P* = 0.0007 and ^&^*P* = 0.0196, respectively), *C. duckei* (^&^*P* = 0.0342 and ^&^*P* = 0.0003, respectively), *C. paupera* (64 µg/mL; ^&^*P* = 0.0342) and *C. pubiflora* (^&^*P* = 0.0143 and ^&^*P* = 0.0023, respectively) is presented in Fig. 3B. On the other hand, pre-treatment of parasites with SDZ + PYR did not interfere with the number of cells with parasites attached to them, nor with the number of parasites adhered to cells in comparison to controls (Fig. 3A,B). Representative images highlighting the impact of oleoresin treatments (64 µg/mL) in the tachyzoites-host cell interaction are in Fig. 3C–H.

**Figure 4:**
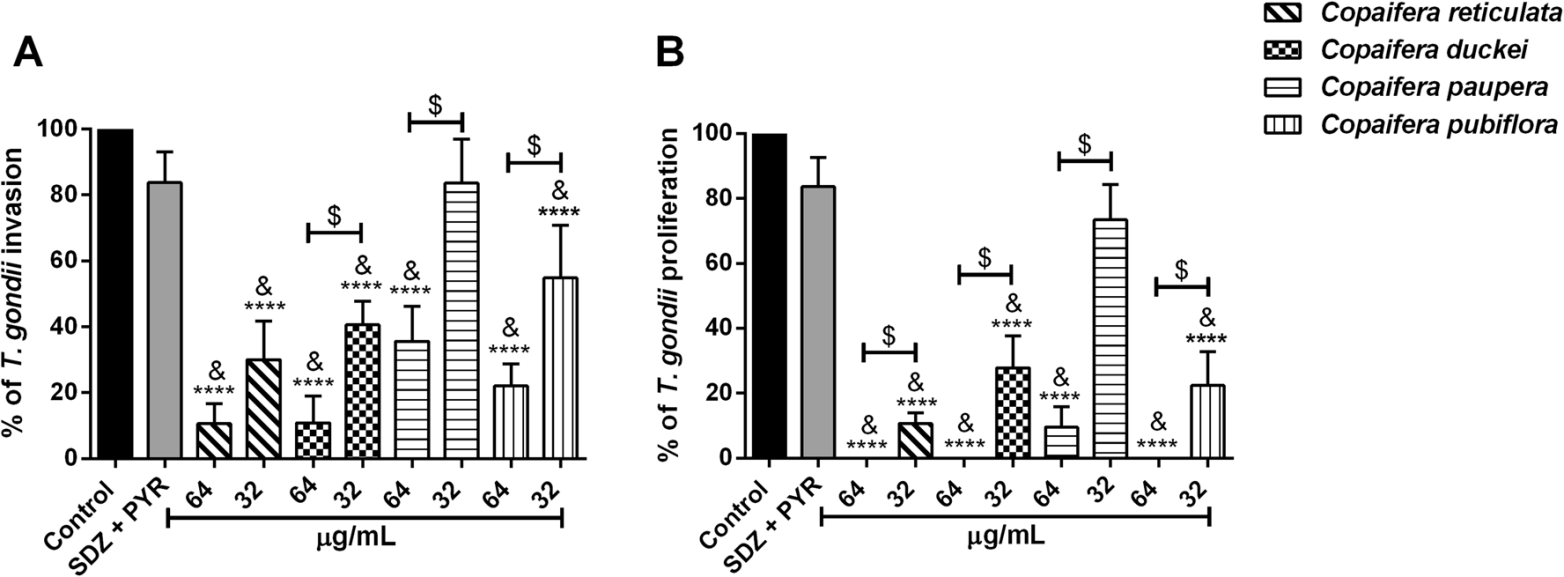
Invasion and intracellular proliferation of pre-treated *T. gondii* tachyzoites in BeWo cells*. T. gondii* tachyzoites were pre-incubated for one h with 64 or 32 µg/mL of oleoresins from *C. reticulata*, *C. duckei*, *C. paupera*, *C. pubiflora*, SDZ + PYR (200 + 8 μg/mL) or culture medium alone (control group/untreated group). (**A**) % of *T. gondii* invasion and (**B**) % of *T. gondii* intracellular proliferation after 24 h were determined using the β-galactosidase assay. Untreated parasites (control group) were considered as 100% of invasion and proliferation, respectively. Data are expressed as means ± standard deviation from experiments performed in eight replicates. ^*^Comparison between untreated parasites and treated parasites; ^&^Comparison to SDZ + PYR-treated parasites; ^$^Comparison between both treatment concentrations of each *Copaifera* oleoresin. Significant differences were analyzed using one-way ANOVA and Tukey’s multiple comparisons post-test. Differences were considered significant when *P* < 0.05.

In the second set of experiments, we assessed whether oleoresins were able to affect parasite invasion and subsequent intracellular proliferation. Concerning parasite invasion, our data suggest that the pre-treatment of *T. gondii* tachyzoites for one hour with 64 and 32 µg/mL oleoresins from *C. reticulata, C. duckei, C. pubiflora* (^****^P < 0.0001) and *C. paupera* (64 µg/mL; ^****^P < 0.0001) reduced significantly parasite invasion (3 h) compared to control group (Fig. 4A). Also, the pre-treatment with both concentrations of oleoresins was significantly more effective in inhibiting parasite invasion (3 h) when compared to parasites treated with SDZ + PYR. Data from *C. reticulata, C. duckei,* (^&^*P* < 0.0001), *C. paupera* (64 µg/mL; ^&^*P* < 0.0001) and *C. pubiflora* (64 and 32 µg/mL; ^&^*P* < 0.0001 and ^&^*P* = 0.0090, respectively) are shown in Fig. 4A. Moreover, oleoresins from *C. duckei*, *C. pubiflora* (^$^*P* < 0.0001), and *C. paupera* (^$^*P* = 0.0062) in the concentration of 64 µg/mL were more efficient to reduce parasite invasion, in comparison with 32 µg/mL concentration. The pre-treatment with SDZ + PYR did not alter the parasite invasion (Fig. 4A).

Addressing the impact of oleoresins in the intracellular proliferation of *T. gondii* (24 h), the concentrations 64 and 32 µg/mL of oleoresins reduced the replication of pre-treated parasites compared to untreated parasites, as follows: *C. reticulata, C. duckei, C. pubiflora* (^****^P < 0.0001) and *C. paupera* (64 and 32 µg/mL; ^****^P < 0.0001 and ^***^P = 0.0002, respectively) (Fig. 4B). Related to the action of each concentration from selected oleoresin in the inhibition of parasite replication, the pre-treatment with 64 µg/mL oleoresins from *C. reticulata* (^$^*P* < 0.0038), *C. duckei, C. paupera* and *C. pubiflora* (^$^*P* < 0.0001) was able of blocking parasite proliferation when compared to the concentration of 32 µg/mL (Fig. 4B). Similarly, all oleoresins, except *C. paupera* at 32 µg/mL*,* showed inhibition of *T. gondii* proliferation when compared to parasites treated with SDZ + PYR. Data from *C. reticulata, C. duckei, C. paupera,* and *C. pubiflora* (^&^*P* < 0.0001) (Fig. 4B).

### Oleoresins exhibited an irreversible concentration-dependent antiparasitic action

**Figure 5:**
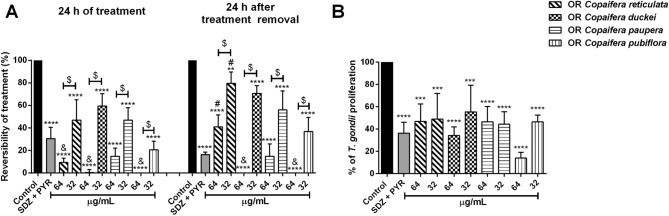
Evaluation of the maintenance of antiparasitic effects of oleoresins by reversibility assay. *T. gondii*-infected BeWo cells were exposed to 64 and 32 µg/mL of oleoresins from *C. reticulata*, *C. duckei*, *C. paupera*, *C. pubiflora*, SDZ + PYR (200 + 8 μg/mL) and culture medium alone (control group/untreated group) for 24 h, followed by treatment removal for an additional 24 h. Parasite proliferation at 24 h of treatment with oleoresins was considered the baseline for comparison. (**A**) The reversibility ratio measures the ability of the parasites to recover from treatment and regain infectiveness. The ratio is shown in percentage change (% reversibility of treatment) in comparison with the control group, which was considered as 100% reversibility. The β-galactosidase assay was used as an indicator of parasite activity. (**B**) Parasites obtained from oleoresin-treated BeWo cells were harvested and used to infect new host cells for 24 h. The number of tachyzoites was determined by β-galactosidase assay and expressed as % of *T. gondii* proliferation. Data are shown as means ± standard deviation from experiments performed in eight replicates. ^*^Comparison between infected/untreated cells and infected/treated cells; ^&^Comparison to SDZ + PYR-infected/treated cells; ^$^Comparison between either treatment concentrations of each species of *Copaifera* oleoresin. ^#^Comparison with the respective treatment in the condition of 24 h of treatment. Significant differences were analyzed using one-way ANOVA and Tukey’s multiple comparisons post-test. Differences were considered significant when *P* < 0.05.

To determine whether the antiparasitic effects promoted by *Copaifera* oleoresins were reversible, we exposed parasite-infected BeWo cells with all four oleoresins for 24 h; rinsed the cell monolayers, and then incubated the cells in the treatment-free medium for a further 24 h. In parallel, we quantified the parasite proliferation at 24 h of treatment with oleoresins; the results served as the baseline for comparison. In agreement with our previous results from the β-galactosidase activity-based screen, all four oleoresins tested in the concentrations of 64 and 32 µg/mL were able to control parasite proliferation after 24 h of treatment, compared to the untreated group (control) (Fig. 5A). Interestingly, the treatment with 64 and 32 µg/mL oleoresins reduced the rate of parasite proliferation even after 24 h of treatment removal in contrast with the untreated group (considered as 100% of reversibility); *C. duckei, C. paupera*, *C. pubiflora* (^****^P < 0.0001), *C. reticulata* (64 and 32 µg/mL; ^****^P < 0.0001 and ^*^P = 0.0099, respectively) and SDZ + PYR (^****^P < 0.0001). It is noteworthy that the treatment with 64 µg/mL oleoresins from *C. duckei* (^&^*P* < 0.0231) and *C. pubiflora* (^&^*P* < 0.0134) were more efficient in the inhibition of parasite growth in comparison with SDZ + PYR treatment. Additionally, the oleoresin concentration of 64 µg/mL from *C. reticulata*, *C. duckei*, *C. paupera,* and *C. pubiflora* (^$^*P* < 0.0001) demonstrated to be more efficient than the concentration of 32 µg/mL (Fig. 5A). Our experiments suggest that treatment with *C. duckei*, *C. paupera*, *C. pubiflora,* and SDZ + PYR did not show significant differences in antiparasitic effect, even after 24 h of treatment removal. Only the treatment with *C. reticulata* (64 and 32 µg/mL; ^#^*P* < 0.0001) tended to be reversible after 24 h. These results suggest that the oleoresins, as well as a combination of SDZ + PYR, maintained their anti-proliferative effect in a concentration-dependent manner, even treatment removal (Fig. 5A).

In order to gain insights into the effect triggered by oleoresins in *T. gondii* tachyzoites, we assessed whether intracellular parasites obtained from oleoresin-treated BeWo cells could sustain their ability to infect new host cells. BeWo cells were infected and treated with *Copaifera* oleoresins for 24 h. Next, the parasites were harvested and used to infect new host cells. We demonstrated that the treatment with 64 and 32 µg/mL oleoresin from *C. reticulata* (^***^P = 0.0001) *C. duckei* (64 and 32 µg/mL; ^****^P < 0.0001 and ^***^P = 0.0008, respectively)*, C. paupera*, *C. pubiflora* (^****^P < 0.0001) and SDZ + PYR (^***^P < 0.0001) were able to impair *T. gondii* infection, compared to parasites from untreated cells (control group) (Fig. 5B). These findings indicate that the inhibitory effects promoted by oleoresins from *C. reticulata*, *C. duckei*, *C. paupera,* and *C. pubiflora*, as well as SDZ + PYR treatment, are irreversible.

### Oleoresin treatments cause cell cycle arrest in parasites in the S/M phase

**Figure 6:**
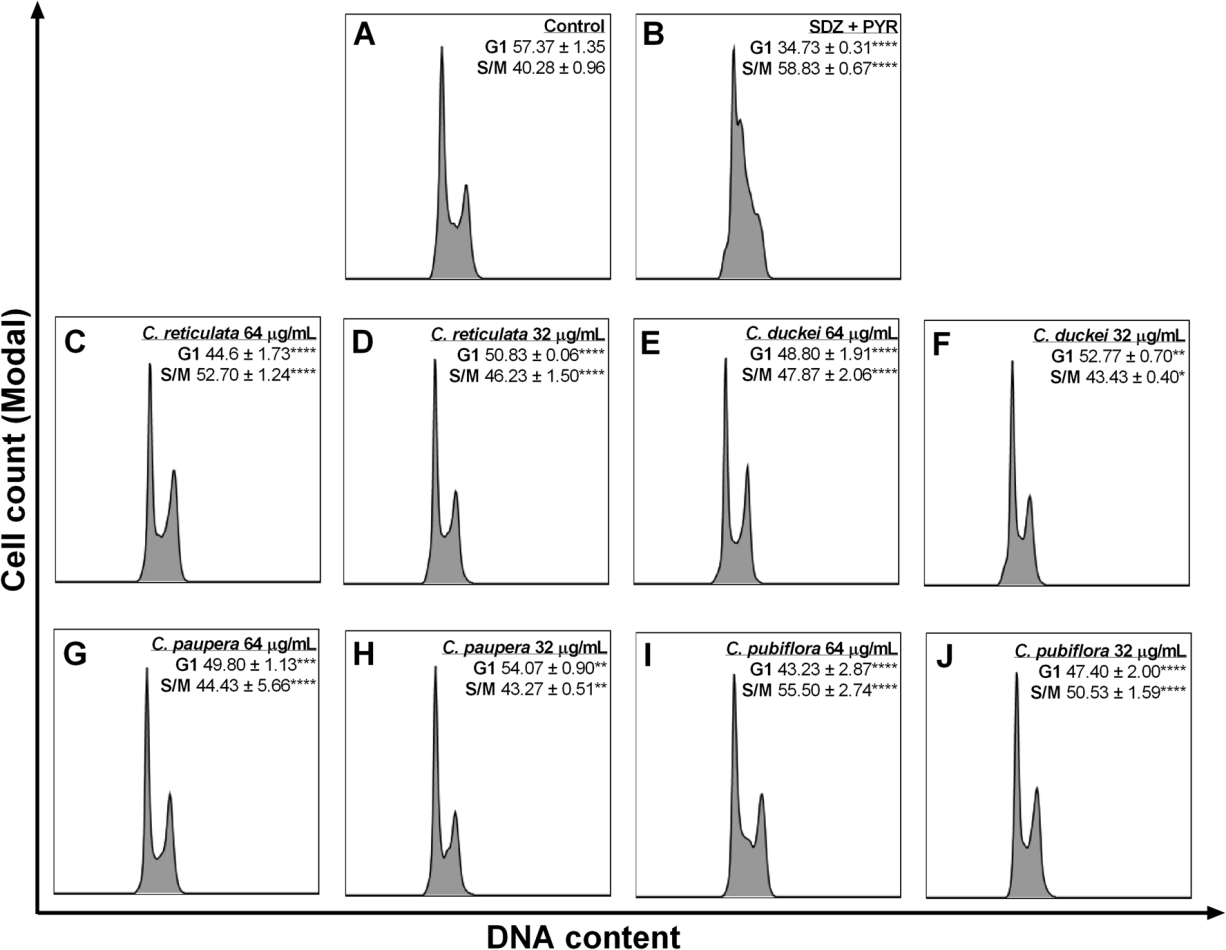
Effects of oleoresins on *T. gondii* cell cycle. Intracellular parasites were exposed for 24 h to either (**A**) culture medium only (control group), (**B**) SDZ + PYR (200 + 8 µg/mL), oleoresins from (**C**) *C. reticulata* 64 µg/mL, (**D**) 32 µg/mL, (**E**) *C. duckei* 64 µg/mL, (**F**) 32 µg/mL, (**G**) *C. paupera* 64 µg/mL, (**H**) 32 µg/mL, (**I**) *C. pubiflora* 64 µg/mL and (**J**) 32 µg/mL. Each representative histogram shows 20.000 total events. Percentage ± standard deviations of parasites in G1 or S/M phases determined by gating is indicated in the accompanying histogram. The experiment was carried out in quadruplicates. ^*^Comparison between infected and untreated cells (control group). Significant differences were analyzed using one-way ANOVA and Tukey’s multiple comparisons post-test. Differences were considered significant when *P* < 0.05.

*Copaifera* oleoresins have an efficient antiparasitic activity by inhibition of parasite proliferation. In this sense, to confirm the anti-proliferative effect showed by these oleoresins, we proposed to evaluate whether progression through the parasite cell cycle was blocked or altered by the oleoresin treatments. First, intracellular parasites were obtained from infected BeWo cells treated for 24 h with oleoresins. Subsequently, we determined the percentages of parasites in each cell cycle phase (G1 and S/M) through the quantification of DNA content by flow cytometry. We observed that the treatment with 64 and 32 µg/mL of oleoresins caused a significant decrease in the 2 N cell number (G1 phase) in the parasite cell cycle compared to untreated parasites in the control group (Fig. 6A). Data from *C. reticulata* (^****^P < 0.0001) (Fig. 6C,D), *C. duckei* (64 and 32 µg/mL; ^****^P < 0.0001 and ^**^P = 0.0014, respectively) (Fig. 6E,F), *C. paupera* (64 and 32 µg/mL; ^****^P < 0.0001 and ^**^P = 0.0023, respectively) (Fig. 6G,H), *C. pubiflora* (^****^P < 0.0001) (Fig. 6I,J) are summarized in the Fig. 6. Oleoresin treatment resulted in significant accumulation in the number of cells in the S/M phase in the parasite cell cycle. Data from 64 and 32 µg/mL of oleoresins from *C. reticulata* (^****^P < 0.0001) (Fig. 6C,D), *C. duckei* (64 and 32 µg/mL; ^****^P < 0.0001 and ^*^P = 0.0210, respectively) (Fig. 6E,F), *C. paupera* (64 and 32 µg/mL; ^****^P < 0.0001 and ^**^P = 0.0023, respectively) (Fig. 6G,H)*, C. pubiflora* (^****^P < 0.0001) (Fig. 6I,J) related to untreated parasites (control group) (Fig. 6A), thus suggesting parasite cell cycle arrest in the S/M phase. To determine the specificity of oleoresin effects on the parasite cell cycle, we assessed the effects of classical antiparasitic drugs (SDZ + PYR). Similarly to oleoresins effects on parasite cell cycle, our results showed that SDZ + PYR treatment caused a decrease in G1 (^****^P < 0.0001) and a prominent accumulation of cells in the S/M phase (^****^P < 0.0001) in comparison with the control group (Fig. 6B).

### Oleoresin treatments promoted ultra-structural alterations in intracellular parasite morphology

**Figure 7:**
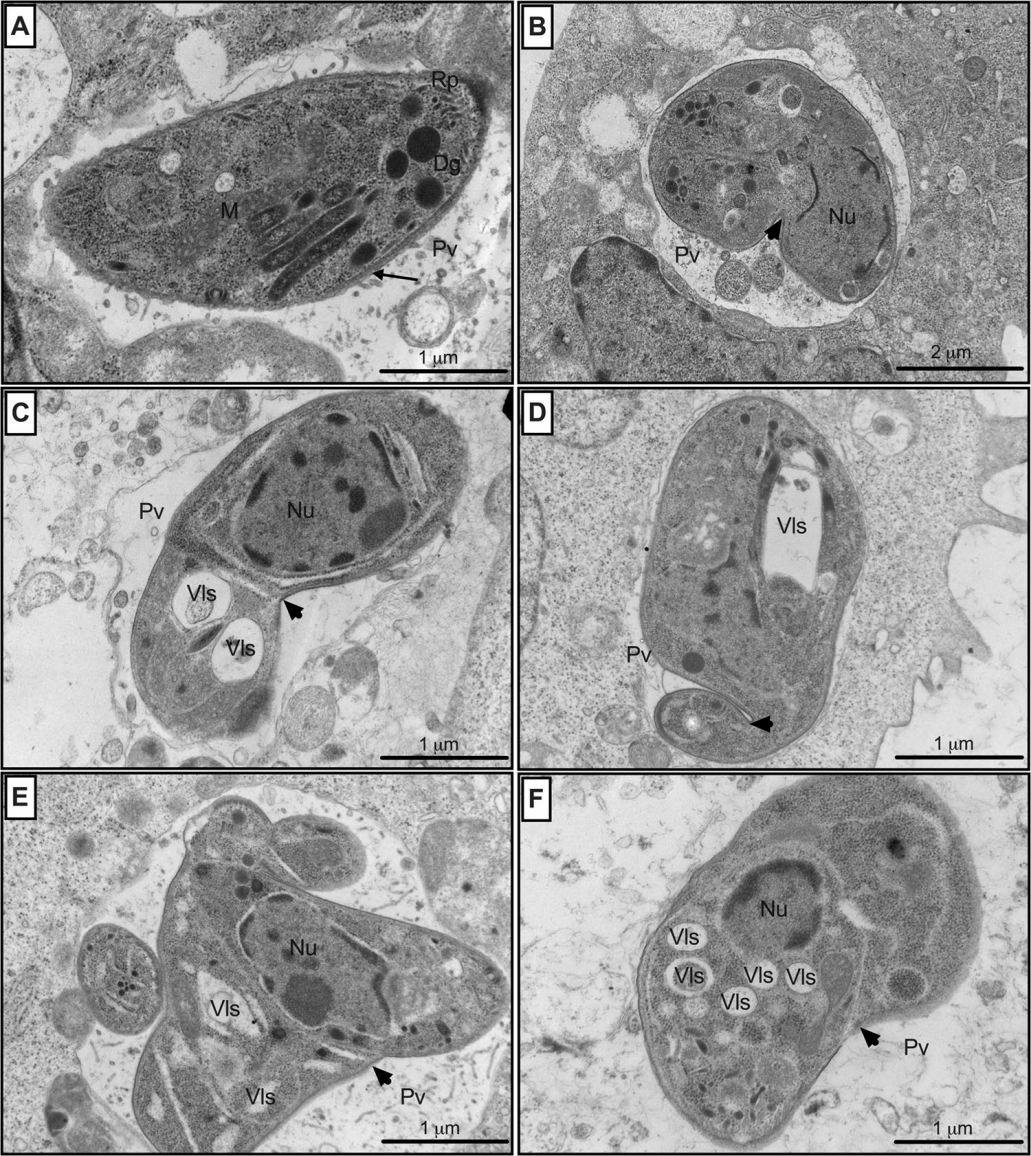
Parasite ultrastructure after exposure to treatments. Transmission electron micrographs of intracellular *T. gondii* after 24 h exposure to either (**A**) culture medium only (control group), (**B**) SDZ + PYR (200 + 8 µg/mL), 64 µg/mL oleoresins from (**C**) *C. reticulata*, (**D**) *C. duckei*, (**E**) *C. paupera* and (**F**) *C. pubiflora*. Pv, parasitophorous vacuole; Rp, rhoptries; Nu, nucleus; Vls, vacuole-like structure; M, mitochondria; and Dg, dense granule of *T. gondii* tachyzoites. Arrow: parasite duple membrane; arrowhead: tethered parasites (basal ends) or single membrane of parasites. Bars scale (bottom right): 1 µm or 2 µm.

We performed the ultrastructural analysis of intracellular *T. gondii* tachyzoites treated with oleoresins to investigate the direct effect of oleoresins in the parasite’s morphology. BeWo cells were infected and treated with 64 µg/mL *Copaifera* oleoresins and SDZ + PYR for 24 h. Next, the infected cells were fixed and processed for transmission electron microscopy (TEM). Untreated cells harbor a parasitophorous vacuole (Pv) containing tachyzoites with a characteristic arc-like shape, a well-defined tubulovesicular network, duple membrane (arrows), rhoptries (Rp), nucleus (Nu), dense granule (Dg) and mitochondria (M), suggesting a normal endogenic process of replication (Fig. 7A). SDZ + PYR-treated infected cells resulted in the disruption of parasite organelles, ruffled duple membranes, as well as parasites united by their basal ends (arrowheads) (Fig. 7B).

Similarly to standard treatment, oleoresin promoted notable alteration in parasite morphology. Infected cells treated with all four oleoresins frequently showed PV containing parasites with multiply intracellular vacuole-like structures (Vls), and tethered tachyzoites with probable difficulty in completing cytokinesis, since the treatment resulted in growth arrest. Also, we visualized the disruption of parasite organelles, as they were poorly defined and reduced to a single membrane structure in some areas, suggesting damage to the formation of the duple membrane (arrowheads) (Fig. 7C–F).

### *Copaifera* oleoresins modulate cytokine release and ROS production by BeWo cells

**Figure 8:**
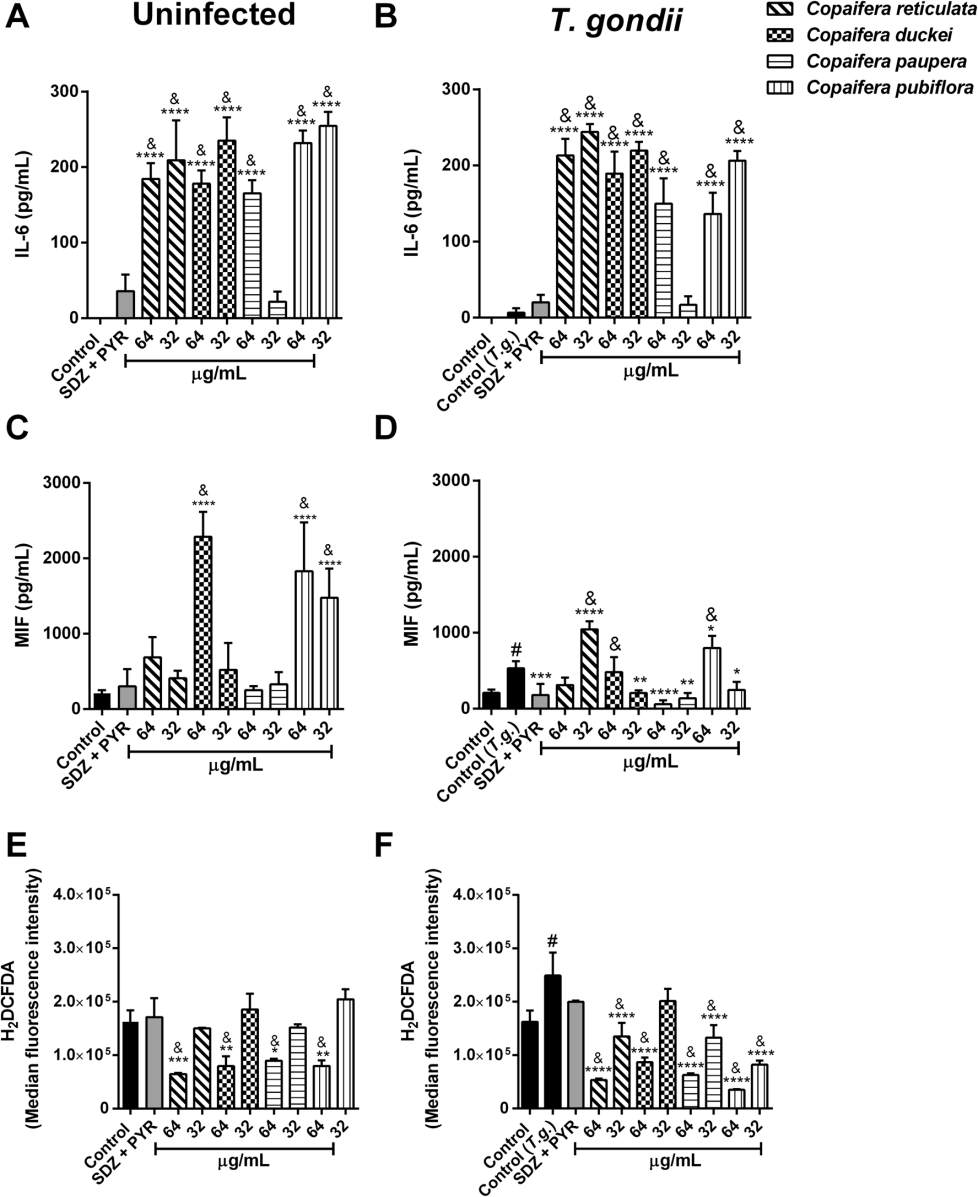
Cytokine and ROS production by *T. gondii*-infected BeWo cells after treatments. BeWo cells were infected or not with *T. gondii* tachyzoites, followed by treatment for 24 h with 64 and 32 µg/mL of oleoresins from *C. reticulata*, *C. duckei*, *C. paupera*, *C. pubiflora*, SDZ + PYR (200 + 8 μg/mL) and culture medium only (control group/untreated group). Supernatants were collected for measurement of (**A**,**B**) IL-6 and (**C**,**D**) MIF. (**E**,**F**) BeWo cells were incubated with the probe 2′,7′-dichlorodihydrofluorescein diacetate (H_2_DCF-DA) and the ROS production was measured by flow cytometry and expressed as median fluorescence intensity (MFI). Data are expressed as means ± standard deviation from experiments performed in five replicates. ^*^Comparison between uninfected/untreated cells (control) and uninfected/treated cells (**A**,**C**,**E**); ^*^Comparison between infected/untreated cells (control *T.g.*) and infected/treated cells (**B**,**D**,**F**); ^#^Comparison between uninfected/untreated cells (control) and infected/untreated cells (control *T.g.*); ^&^Comparison in relation to the SDZ + PYR treatment. Significant differences were determined by using one-way ANOVA and Tukey’s multiple comparisons post-test. Differences were considered significant when *P* < 0.05.

While our previous results described above indicate that *Copaifera* oleoresins have a direct effect on parasites, we could not exclude the possibility that these oleoresins may affect intracellular proliferation by altering the host cell environment. In this context, firstly, we investigated the potential immunomodulatory effects of *Copaifera* oleoresins by measuring the levels of IL-6, MIF, TNF-α, TGF-β, and IL-10 present in the supernatants from BeWo cells infected or not by *T. gondii*. Interestingly, our results showed that in the absence of infection, except for *C. paupera* oleoresin in the concentration of 32 µg/mL, the treatment with 64 and 32 µg/mL of oleoresins from *C. reticulata*, *C. duckei*, *C. paupera,* and *C. pubiflora* (^****^P < 0.0001; ^&^*P* < 0.0001) induced an increase in the levels of IL-6 in comparison to controls. Additionally, SDZ + PYR treatment did not interfere in the IL-6 production (Fig. 8A). Similarly, even after *T. gondii* infection, except for *C. paupera* oleoresin in the concentration of 32 µg/mL, infected BeWo cells treated with 64 and 32 µg/mL of oleoresins from *C. reticulata*, *C. duckei*, *C. paupera* and *C. pubiflora* (^****^P < 0.0001; ^&^*P* < 0.0001) continued producing high levels of IL-6, compared to infected and untreated cells (control group) and SDZ + PYR-treated cells. Moreover, *T. gondii* infection (control *T.g.*) and the SDZ + PYR standard treatment did not alter the IL-6 production in comparison with uninfected and untreated cells (control) (Fig. 8B). Addressing MIF production, we observed that only the treatment with 64 µg/mL *C. duckei* oleoresin (^****^P < 0.0001; ^&^*P* < 0.0001), 64 and 32 µg/mL *C. pubiflora* oleoresin (^****^P < 0.0001; ^&^*P* < 0.0001) caused an augmentation of MIF levels by uninfected BeWo cells in comparison with uninfected and untreated cells (control group) and SDZ + PYR-treated cells (Fig. 8C). Curiously, *T. gondii* infection increased MIF production by BeWo cells related to uninfected and untreated cells (^#^*P* = 0.0030). Our results showed that the treatment of infected cells only with oleoresins from 32 µg/mL *C. reticulata* (^****^P < 0.0001) and 64 µg/mL *C. pubiflora* (^*^P = 0.0270) up-regulated the MIF levels in comparison to infected and untreated cells (Fig. 8D). In contrast, oleoresins from 32 µg/mL *C. duckei* (^**^P = 0.0032), 64 and 32 µg/mL *C. paupera* (^****^P < 0.0001 and ^**^P = 0.0014, respectively), 32 µg/mL *C. pubiflora* (^*^P = 0.0139) and SDZ + PYR treatment (^***^P = 0.0009) down-modulated the MIF production, when compared to infected and untreated cells (control *T.g.*) (Fig. 8D). Besides, the treatment with oleoresins from 32 µg/mL *C. reticulata* (^&^*P* < 0.0001), 64 µg/mL *C. duckei* (^&^*P* = 0.0069) and 64 µg/mL *C. pubiflora* (^&^*P* < 0.0001) led to the infected cells to produce more MIF that the cells treated with SDZ + PYR (Fig. 8D). TNF-α, TGF-β, and IL-10 cytokines were not detected in supernatants from BeWo cells under any experimental conditions (data not shown).

Moreover, we also investigated the effects of *Copaifera* oleoresins in ROS production by BeWo cells. Our data indicates that in the absence of *T. gondii* infection, the treatment with 64 µg/mL of oleoresins from *C. reticulata* (^***^P = 0.0002; ^&^*P* < 0.0001), *C. duckei* (^**^P = 0.0012; ^&^*P* = 0.0004), *C. paupera* (^*^P = 0.0133; ^&^*P* = 0.0047) and *C. pubiflora* (^**^P = 0.0013; ^&^*P* = 0.0004) caused a downregulation in ROS production in relation to uninfected and untreated cells (control group) and SDZ + PYR-treated cells (Fig. 8E). Also, *T. gondii* infection (control *T.g*.) promoted an increase in ROS levels related to uninfected and untreated cells (control group) (^#^*P* = 0.0007). Interestingly, except for *C. duckei* oleoresin in the concentration of 32 µg/mL, the treatment with 64 and 32 µg/mL of oleoresins from *C. reticulata*, *C. duckei*, *C. paupera* and *C. pubiflora* (^****^P < 0.0001) was able to reduce the ROS levels caused by *T. gondii* infection (Fig. 8F). Furthermore, except for *C. duckei* oleoresin in the concentration of 32 µg/mL, the reduction in ROS production was significantly higher in the treatments with oleoresins from *C. reticulata* (64 and 32 µg/mL; ^&^*P* < 0.0001 and ^&^*P* = 0.0173, respectively), *C. duckei* (64 µg/mL; ^&^*P* < 0.0001), *C. paupera* (64 and 32 µg/mL; ^&^*P* < 0.0001 and ^&^*P* = 0.0125, respectively) and *C. pubiflora* (64 and 32 µg/mL; ^&^*P* < 0.0001) in comparison with cells infected and treated with SDZ + PYR (Fig. 8F). Finally, SDZ + PYR treatment did not affect the ROS production in both the absence and presence of infection (Fig. 8E,F).

### Human chorionic villous explants treated with oleoresins altered viability only at the highest concentration

**Figure 9:**
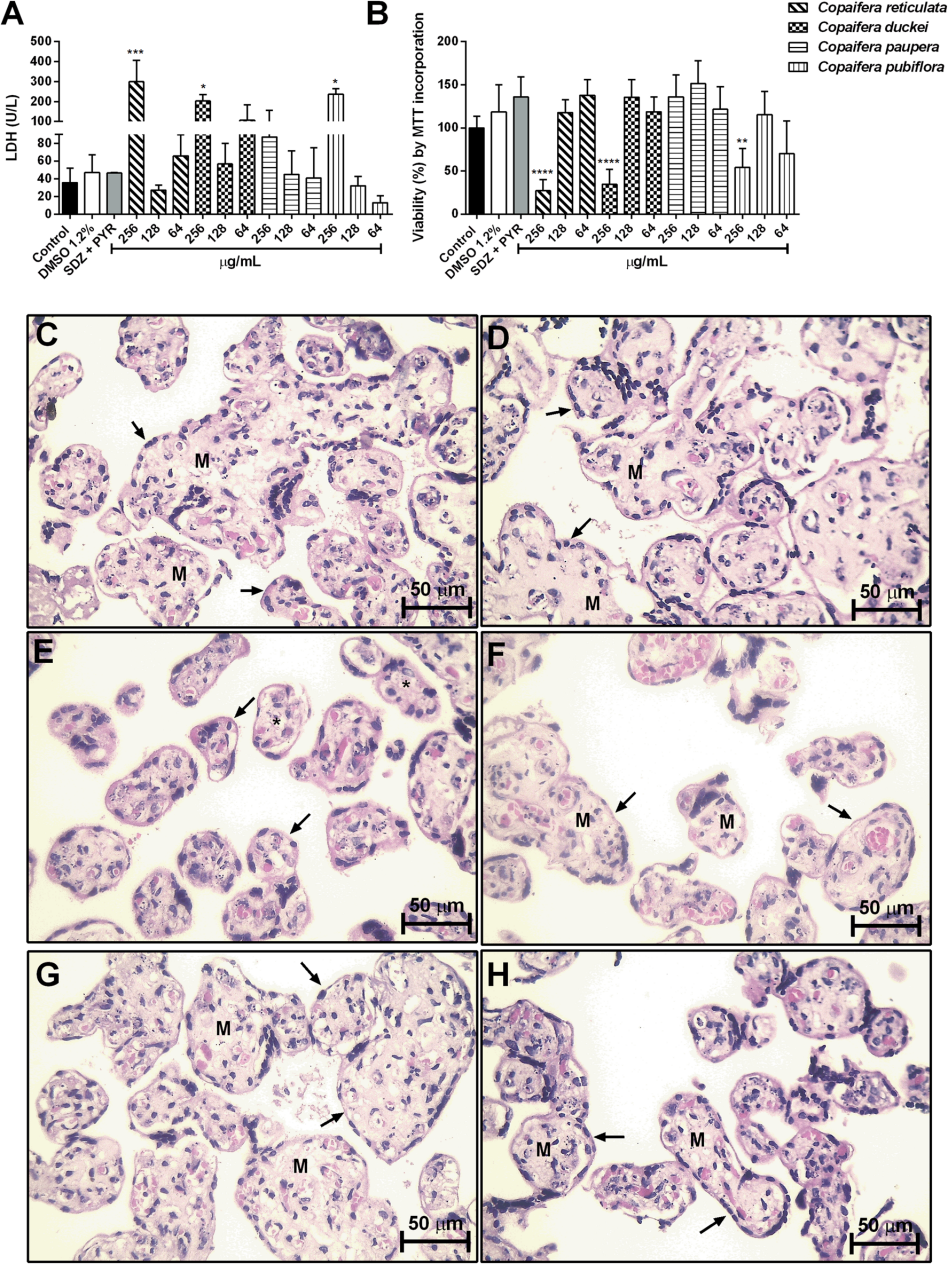
Viability of human villous explant after treatments. Villous explants were treated for 24 h with high oleoresin concentrations of *C. reticulata*, *C. duckei*, *C. paupera* and *C. pubiflora* (256, 128 and 64 µg/mL), SDZ + PYR (150 and 200 µg/mL), DMSO (1.2%) and culture medium alone (control group/untreated group). (**A**) Supernatants were collected and used to measure LDH (U/L) levels. (**B**) MTT assays with villous explants and the tissue viability are shown in percentages (viability % by MTT incorporation). Representative photomicrographs of villi incubated with (**C**) culture medium alone (control group), (**D**) SDZ + PYR, (**E**) *C. reticulata*, (**F**) *C. duckei*, (**G**) *C. paupera* and (**H**) *C. pubiflora* are shown in this figure. Representative images of oleoresin treatments are shown only at the concentration of 128 µg/mL. Data are expressed as means ± standard deviation from experiments performed in eight replicates. ^*^Comparison between untreated cells and treated cells. Significant differences were analyzed by the Kruskal–Wallis test and Dunn’s multiple comparison post-test (statistically significant when *P* < 0.05). Hematoxylin–eosin (HE) stained histological sections show Syncytiotrophoblast cells (black arrows) and mesenchyme (M). Scale bar: 50 µm.

In addition to investigating the effects of *Copaifera* oleoresins in *T. gondii*-infected BeWo cells, we also evaluated the action of these oleoresins against parasite infection in human placental villous explants. Firstly, to determine a non-toxic concentration to use in the further set up of experiments, the viability of villous explants after oleoresin treatments was performed by measuring LDH release and MTT assay. Our data demonstrated that the treatment only in the concentration of 256 µg/mL oleoresins from *C. reticulata* (^***^P = 0.0004), *C. duckei* (^*^P = 0.0136), and *C. pubiflora* (^*^P = 0.0226) caused a significant increase in the release of LDH (U/L) by villous explants when compared to untreated villous explants (control group);. In contrast, *C. paupera* oleoresins did not alter the LDH levels at all concentrations tested (Fig. 9A). Also, the treatment with SDZ + PYR and DMSO 1.2% did not cause significant cytotoxicity in villous explants compared to untreated villous explants (control group) (Fig. 9A). Similar to LDH measurements, our results from MTT incorporation showed a significant reduction in the viability of villous explants only at 256 µg/mL of oleoresins from *C. reticulata*, *C. duckei* (^****^P < 0.0001), and *C. pubiflora* (^**^P = 0.0073) compared to untreated controls; while all others experimental conditions did not alter tissue integrity (Fig. 9B). Based on these results, we established the use of 128 µg/mL as the highest concentration that did not change the tissue integrity at all four oleoresins for further experiments. We analyzed the morphology of the villus explants to corroborate with the data from the viability assay. Our results demonstrated that the oleoresin treatments did not alter the tissue morphology, which was highlighted by the typical morphology of the syncytiotrophoblast cells (black arrows) and mesenchyme (M) when compared to untreated villous explants (Fig. 9C–H).

### *Copaifera* oleoresins can control *T. gondii* intracellular proliferation in human villous explants

**Figure 10:**
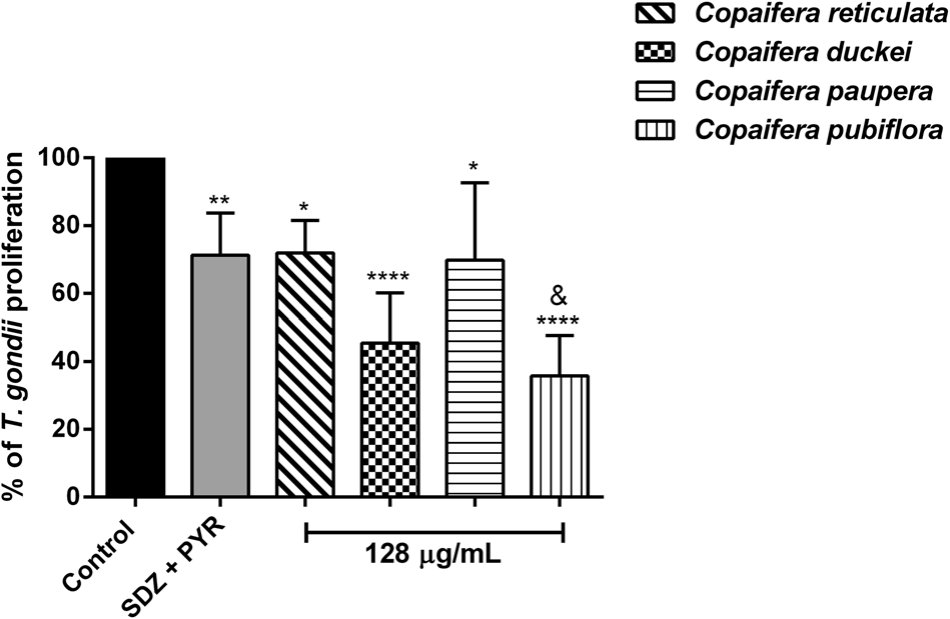
*T. gondii* intracellular proliferation in infected-villous explants. Human villous explants were infected with *T. gondii* tachyzoites for 24 h, followed by treatment for additional 24 h with 128 µg/mL of oleoresins from *C. reticulata*, *C. duckei*, *C. paupera*, *C. pubiflora*, SDZ + PYR (150 + 200 μg/mL) and culture medium alone (control group/untreated group). *T. gondii* intracellular proliferation in villous explants was measured by β-galactosidase assay, and the number of tachyzoites was expressed in percentage (% of *T. gondii* proliferation), which the untreated/infected (control group) was considered as 100% of parasite proliferation. Data are expressed as means ± standard deviation from experiments performed in five replicates. ^*^Comparison between infected/untreated cells and infected/treated cells; ^&^Comparison in relation to SDZ + PYR-infected/treated cells. Significant differences were determined by using One-way ANOVA and Sidak’s multiple comparisons post-test. Differences were considered significant when *P* < 0.05.

We evaluated the effects of oleoresins from *C. reticulata*, *C. duckei*, *C. paupera,* and *C. pubiflora* (128 µg/mL) in the control of *T. gondii* intracellular proliferation in villous explants using the β-galactosidase assay. We observed that the treatment with all four oleoresins in the concentration of 128 µg/mL significantly reduced the percentage of intracellular parasites, as follow: *C. reticulata* (~ 30%; ^*^P = 0.0339), *C. duckei* (~ 60%; ^****^P < 0.0001), *C. paupera* (~ 30%; ^*^P = 0.0404) and *C. pubiflora* (~ 65%; ^****^P < 0.0001) compared to untreated villous explants (control group—considered as 100% of parasite proliferation). Interestingly, the treatment with 128 µg/mL *C. pubiflora* oleoresin (^&^*P* = 0.0056) was more efficient in the control of parasite replication than the SDZ + PYR treatment (Fig. 10). As expected, the treatment with SDZ + PYR (150 and 200 µg/mL, respectively) also inhibited parasite growth (~ 30%; ^**^P = 0.0093) compared to untreated villous explants (Fig. 10). Additionally, we also tested whether the concentration of 64 µg/mL (non-toxic concentration) of all oleoresins used in this work would be able to inhibit parasite growth in villous explants. However, we observed that only the *C. pubiflora* oleoresin was capable of reducing *T. gondii* tachyzoites proliferation in comparison with untreated villous explants (data not shown).

### Oleoresin treatments down-modulated the TNF-α production in *T. gondii*-infected villous explants

**Figure 11:**
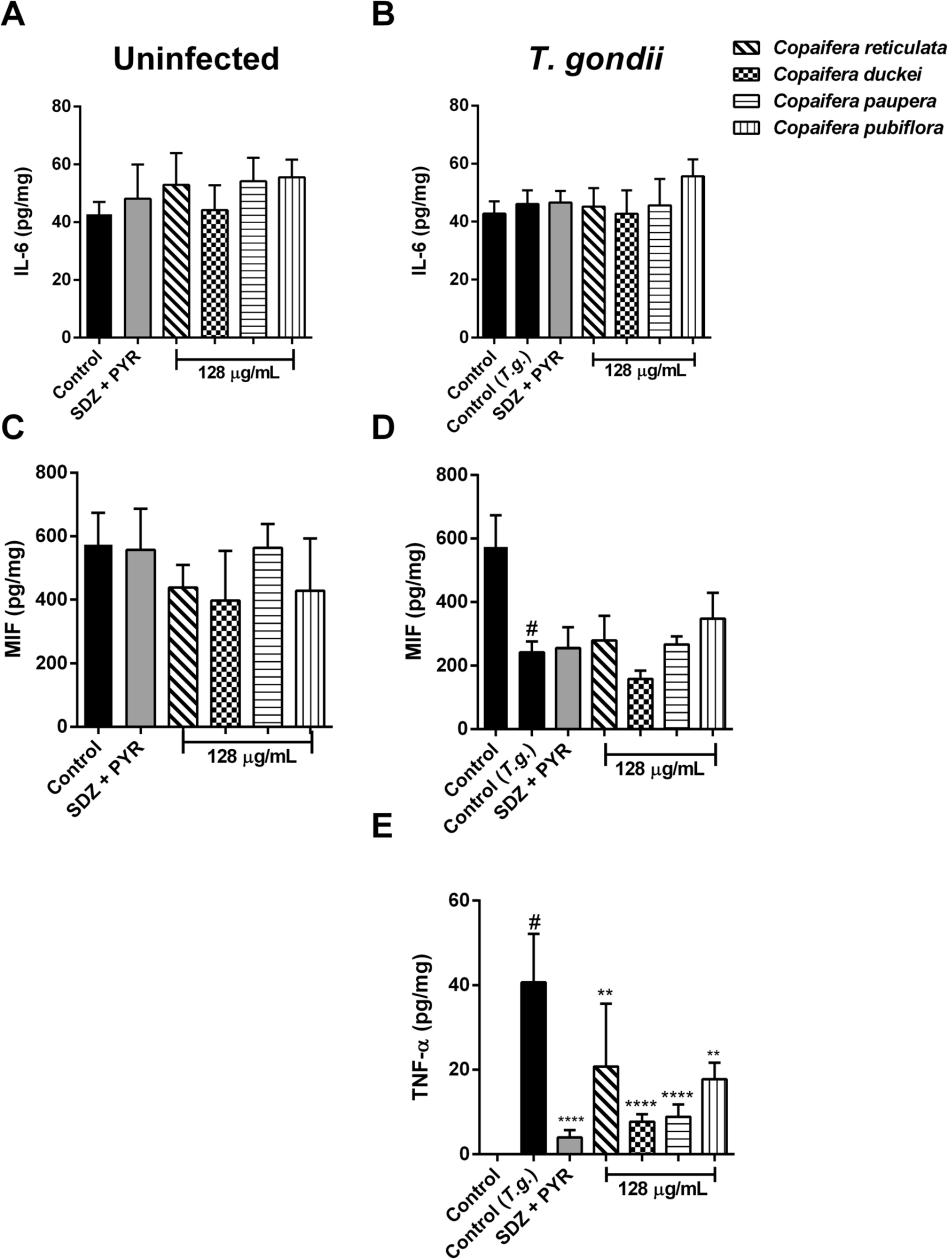
Cytokine production by *T. gondii*-infected human villous explants after treatments. Human villous explants were infected or not with *T. gondii* tachyzoites for 24 h, followed by treatment for additional 24 h with 128 µg/mL of oleoresins from *C. reticulata*, *C. duckei*, *C. paupera*, *C. pubiflora*, SDZ + PYR (150 + 200 μg/mL) and culture medium only (control group/untreated group). Supernatants were collected for measurement of (**A**,**B**) IL-6; (**C**,**D**) MIF and (**E**) TNF-α. Cytokine levels were expressed in pg/mg of tissue. Data are shown as means ± standard deviation from experiments performed in five replicates. ^*^Comparison between uninfected/untreated cells (control) and uninfected/treated cells (**A**,**C**); ^*^Comparison between infected/untreated cells (control *T.g.*) and infected/treated cells (**B**,**D**,**E**); ^#^Comparison between uninfected/untreated cells (control) and infected/untreated cells (control *T.g.*); Significant differences were analyzed using one-way ANOVA and Tukey’s multiple comparisons post-test. Differences were considered significant when *P* < 0.05.

In order to gain insights into the action of oleoresins on the physiology of villous explants, we evaluated whether oleoresin treatments modulate cytokine production profile by measuring the levels of IL-6, MIF, TNF-α, TGF-β and IL-10 present in the supernatants from villous explants infected or not by *T. gondii*. In the absence of infection, the treatment with all four oleoresins and SDZ + PYR did not alter IL-6 and MIF production in comparison to uninfected and untreated villous explants (control group) (Fig. 11A,C). *T. gondii* infection (control *T.g.* group) did not alter the IL-6 production but promoted a reduction of MIF levels compared to uninfected and untreated villous (control group) (^#^*P* < 0.0001). Oleoresins and SDZ + PYR treatment did not change the release of IL-6 or MIF when compared with untreated and infected villous explants (control group) (Fig. 11B,D). Moreover, TNF-α production was not detected in supernatants from uninfected villous explants under any experimental conditions (data not shown). *Toxoplasma gondii* infection (control *T.g.*) caused a sharp increase in the TNF-α levels related to uninfected and untreated villous (control group) (^#^*P* < 0.0001). Interestingly, parasite-infected villous explants treated with 128 µg/mL oleoresins from *C. reticulata* (^**^P = 0.0091), *C. duckei*, *C. paupera* (^****^P < 0.0001), *C. pubiflora* (^**^P = 0.0022) and SDZ + PYR treatment (^****^P < 0.0001) showed a significant reduction in TNF-α release, when compared to untreated and infected villous explants (control group) (Fig. 11E). TGF-β and IL-10 cytokines were not detected in supernatants from villous explants under any experimental conditions (data not shown).

### Proposed model of the effects triggered by *Copaifera* spp. oleoresins during *T. gondii* infection in both BeWo cells and human villous explants

**Figure 12:**
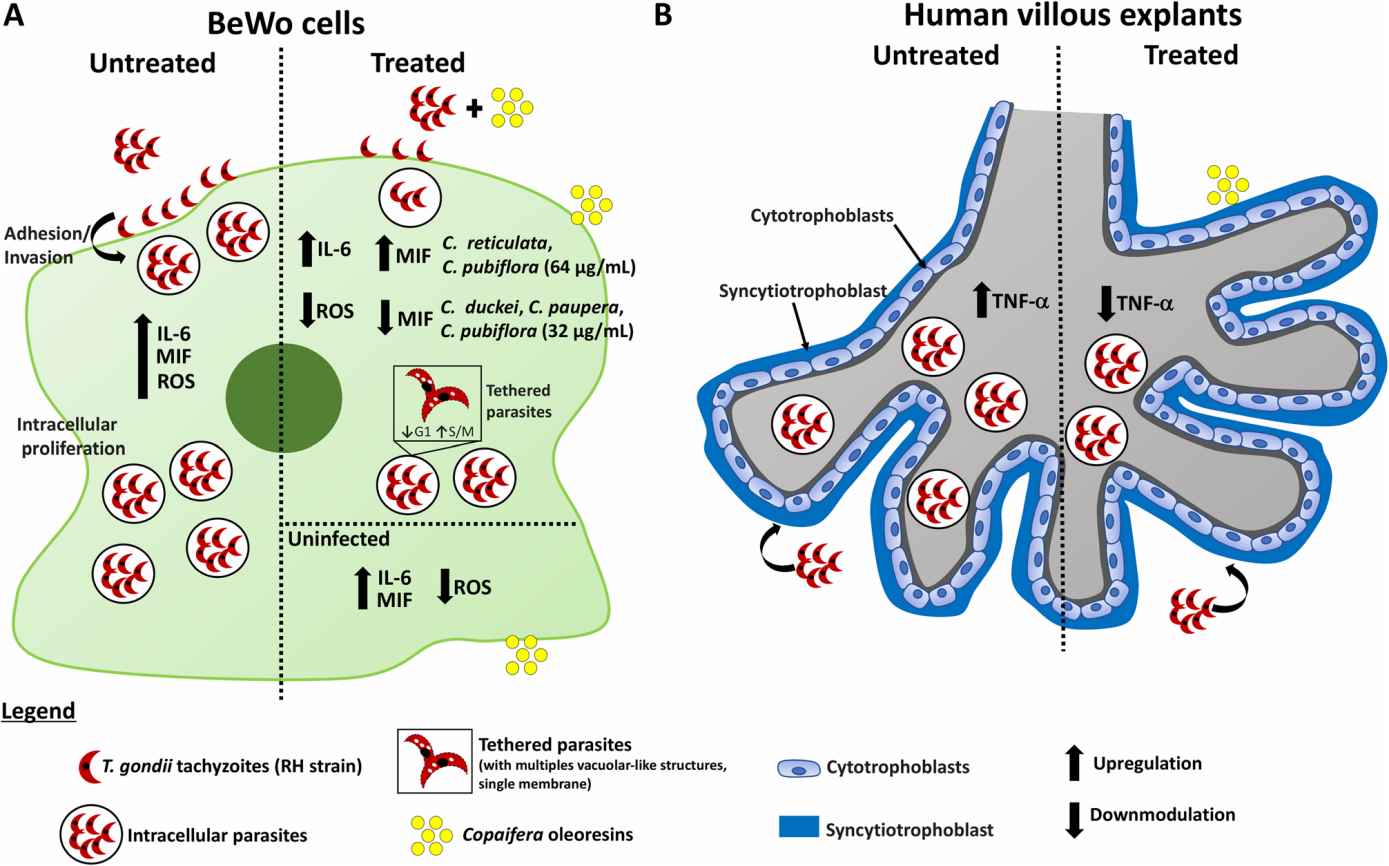
Scheme summarizing the effects of *Copaifera* spp. oleoresins against *T. gondii* infection in both human trophoblastic cells (BeWo cells) and human villous explants. (**A**) The previously pre-treatment of *T. gondii* tachyzoites with *Copaifera* oleoresins (*C. reticulata*, *C. duckei*, *C. paupera,* and *C. pubiflora*) promoted a reduction in the adhesion, invasion and the subsequent proliferation of parasites in BeWo cells. Oleoresin treatments were capable of modulating the cytokine on uninfected BeWo cells by increasing levels of IL-6 and MIF, and a reduction in the ROS production. Moreover, after *T. gondii* infection, BeWo cells responded with increasing the levels of IL-6, MIF, and ROS. In contrast, the oleoresin treatments of infected BeWo cells culminated in control of parasite proliferation with a downmodulation of MIF (oleoresins from *C. duckei*, *C. paupera* and *C. pubiflora*) and ROS, and an upregulation of MIF (oleoresins from *C. reticulata* and *C. pubiflora*) and IL-6. Besides, the impairment in the proliferation of intracellular parasites treated by oleoresins was confirmed by the parasite cell cycle arrest (reduction in G1 phase and an increase in S/M phase), which was evidenced by parasites bound by their basal ends, with multiples vacuolar-like structures and a single membrane in some areas. (**B**) *T. gondii* is capable of infecting and proliferating in human villous explants (crossing the placental barrier: syncytiotrophoblast and cytotrophoblasts), promoting an increase in the concentrations of TNF-α. On the other hand, the oleoresin treatments were able to control intracellular parasite proliferation with a reduction of the TNF-α level.

Based on our results, a proposed model of the mechanism of action of oleoresins against *T. gondii* in both BeWo cells and human villous explants is shown (Fig. 12). Our data showed that previously pre-treatment of *T. gondii* tachyzoites with *Copaifera* oleoresins were able to reduce *T. gondii* adhesion, invasion, and intracellular proliferation in BeWo cells. Also, oleoresin treatments exhibited an irreversible concentration-dependent antiparasitic action and caused a parasite cell cycle arrest in the S/M phase, as well as promoted ultra-structural alterations in the morphology of intracellular parasites. Interestingly, the oleoresin treatments were capable of modulating the cytokine production on uninfected BeWo cells by an augmentation of IL-6 and MIF levels, and a reduction in the ROS production. Moreover, after *T. gondii* infection, BeWo cells responded with increasing the levels of IL-6, MIF, and ROS. In contrast, oleoresins-treated infected BeWo cells culminated in control of parasite proliferation with a downmodulation of MIF (oleoresins from *C. duckei*, *C. paupera* and *C. pubiflora*) and ROS, and an upregulation of MIF (oleoresins from *C. reticulata* and *C. pubiflora*) and IL-6 (Fig. 12A). As shown in BeWo cells, *T. gondii* is capable of infecting and proliferating in human villous explants, inducing an increase in TNF-α concentration. On the other hand, oleoresin treatments were able to control intracellular parasite proliferation with a reduction of the TNF-α level (Fig. 12B). Taken together, we suggest that the anti-*T. gondii* effects triggered by these oleoresins are probably related to the immunomodulation of the host cells and mainly by a direct action on parasites, highlighted by the block of parasite cell cycle progression and ultra-structural alterations, which may compromise the parasite viability.

## Discussion

*Toxoplasma gondii* is the causative agent of toxoplasmosis, one of the most important and challenging diseases in public health^1^. In the context of congenital transmission, when a woman gets the infection during pregnancy, recommended drugs for treatment are limited to a combination of sulfadiazine plus pyrimethamine (SDZ + PYR) and spiramycin^8,19,20,24^. Unfortunately, the use of these drugs has been associated with severe side effects and uncommon reactions in both mother and child^3,27,56^. Additionally, several independent studies have shown that *T. gondii* has an exceptional adaptive potential, which has led to the development of drug-resistant parasites^56–58^. Therefore, it is clear the urgency of studies that focus on finding compounds that are both efficacious and non-toxic for the treatment of congenital toxoplasmosis.

Plant-based compounds are promising alternative treatments for various parasitic diseases, and some have shown antiparasitic effects against *T. gondii* in vitro and in vivo. These compounds may also have fewer side effects when compared to standard drug treatment for congenital toxoplasmosis^47,59^. In this sense, the present study investigated the effects of four oleoresins from different species of *Copaifera* genus (*C. reticulata*, *C. duckei*, *C. paupera,* and *C. pubiflora*) in *T. gondii* infection of human trophoblastic cells (BeWo cells; in vitro model) and human villous explants from the third trimester of pregnancy (ex vivo model). While several studies have demonstrated the antiparasitic effects of *Copaifera* oleoresins^60–64^, this is the first study showing the activity of these oleoresins against *T. gondii*.

In order to investigate the impact of *Copaifera* oleoresins in the control of parasite proliferation, we firstly verified the viability of BeWo cells treated with different concentrations of oleoresins. Our results showed that oleoresins-treated BeWo cells exhibited altered cell viability only at the highest concentrations (256 and 128 µg/mL). Following, we treated *T. gondii*-infected BeWo cells for 24 h with different non-toxic concentrations of each oleoresin. Interestingly, we observed that *Copaifera* oleoresins were as effective as SDZ + PYR treatment in reducing the intracellular proliferation of a highly virulent strain (RH) of *T. gondii* in BeWo cells. Interestingly, all oleoresins tested were able to control parasite proliferation, at the highest concentrations, more efficiently than SDZ + PYR treatment. Curiously, *C. pubiflora* oleoresin was the only compound that inhibited parasite growth at all concentrations used.

*Toxoplasma gondii* is an obligate intracellular protozoan parasite capable of infecting a broad range of nucleated cells. Thus, successful infection the parasites to attach, invade, and proliferate inside host cell^36,55^. Hence, the next question was: would the treatment of *T. gondii* tachyzoites with *Copaifera* oleoresins be able to interfere in essential processes for the perpetuation of the parasite infection? Thus, we set out to assess the impact of oleoresin treatments in adhesion, invasion, and proliferation of the parasites. The pre-treatment of tachyzoites for 1 h with all four oleoresins inhibited parasite attachment (with a decrease in the number of BeWo cells containing adhered parasites and in the number of parasites attached to the cells). Oleoresins also inhibit invasion (observation at 3 h after infection showed a decrease in the percentage of invading parasites) and subsequent proliferation (quantification at 24 h after infection showed a decrease in the percentage of intracellular parasites). In addition, our results showed that the pre-treatment of the parasites with SDZ + PYR did not alter adhesion, invasion, and proliferation processes in vitro. Curiously, Castro-Filice et al*.* (2014)^30^ demonstrated that the pre-treatment of the parasites with a combination of PYR, SDZ, and folinic acid for 1 h reduced *T. gondii* proliferation after 72 h in human villous explants. In comparison with the literature, we conclude in our findings that pre-treatment of parasites with the standard drugs is insufficient to reduce parasitism, at least, during the early stages of infection.

Taken together, we observed that oleoresins and SDZ + PYR exposure seem to affect the parasites in the model of *T. gondii*-infected BeWo cells and the pre-treatment. Thus, we suggest that oleoresin treatments are likely targeting the parasites directly, which may be interfering in essential parasite functions. This hypothesis is only in part, supported by the reversibility assay. Here, we had demonstrated that, after 24 h of treatment, when the oleoresin treatments were removed, the parasites did not recover their proliferation capacity, evidencing that oleoresin treatments were able to promote a powerful blockage in the parasite physiology. Moreover, SDZ + PYR treatment also promoted an irreversible effect in parasite proliferation. Interestingly, oleoresins from both *C. duckei* and *C. pubiflora* (64 µg/mL) showed almost complete ablation of parasite proliferation with 24 h of treatment, and their effects persisted after treatment removal. This data suggests that oleoresins are more efficient to block the recovery of the parasite growth than SDZ + PYR treatment. To corroborate these findings, we exposed parasites to the oleoresin treatments after infection of BeWo cells; the recovered parasites were evaluated for the ability to reinfect new host cells. We showed that the treatment with all four oleoresins at all concentrations impaired *T. gondii* reinfection. In addition, SDZ + PYR treatment promoted similar results when compared to oleoresin treatments.

In summary, we hypothesized that *Copaifera* oleoresins could bind directly in extracellular parasites and are also able to cross the host cell membrane and reach the intracellular parasites. The binding of oleoresins to the parasite membrane may be leading to the morphological alteration in parasite’s structures, and that relates to the impairment of infection by *T. gondii*. Izumi and colleagues partially corroborate the findings shown in this study (2013)^49^; the authors report the effect of eight different species of *Copaifera* oleoresins in the growth inhibition of all life cycle forms of *Trypanosoma cruzi*; showing that the intracellular forms were the most susceptible to treatment. Moreover, these authors also demonstrated that the trypanocidal activity of these oleoresins is mediated by lipid peroxidation and permeability changes in the parasite’s cellular and mitochondrial membranes, leading to high production of reactive oxygen species that can cause parasite damage and culminate in apoptosis^65,66^. Another study showed that *C. paupera* oleoresin was also able to eliminate the two evolutive forms of *Leishmania amazonensis* similarly, as shown against *T. cruzi*^60^. Furthermore, there is one report of *C. reticulata* oleoresin showing antimicrobial activity against *Plasmodium falciparum*, reducing parasitemia levels of infected animals, and showing low cytotoxicity effects^50^. Thus, multiple reports in literature reinforce the concept that *Copaifera* oleoresins are a promising source of compounds with notable antiparasitic effects^67^.

Based on the current literature, we investigated potential mechanisms by which *Copaifera* oleoresins affect *T. gondii* proliferation directly. We tested whether oleoresin treatments blocked or altered parasite cell cycle progression by the quantification of DNA content using flow cytometry techniques. We observed that all four oleoresin treatments at all concentrations caused a significant decrease in the number of parasites in G1 and accumulation in the S/M phase of the parasite cell cycle. Similarly, SDZ + PYR treatment also promoted a reduction in the G1 phase and a strong arrest in S/M phase. These data are in agreement with Lavine and Arrizabalaga (2011)^68^, who demonstrated that the treatment with a low dose of pyrimethamine altered the cell cycle of *T. gondii* by decreasing the number of parasites in G1 phase and promoting a loss of late S/M phase peak, potentially as a result of degradation of DNA. In contrast with the literature, our results suggest disruption of the parasite’s cell cycle (G1 reduction and arrest in S/M phase) in the SDZ + PYR group; which can be explained by the fact that we used the combination of PYR and SDZ and a concentration of PYR about four-fold higher than used by the authors mentioned above. Our data and literature reports suggest that a possible mechanism of action for oleoresins is to arrest parasites in the S/M phase, as has been shown with other compounds that inhibit *T. gondii* growth^68–70^.

Lavine and Arrizabalaga (2011)^68^ showed that monensin, a polyether ionophore antibiotic that inhibits *T. gondii* replication, increases the number of parasites in the S/M phase of the cell cycle, maybe as a consequence of DNA damage or cellular stress. Furthermore, another study suggested that the cell cycle arrest in S/M caused by monensin in *T. gondii* would serve as a signal to initiate an autophagy-like cell death^70,71^. Our results suggest that oleoresin treatments blocked progression trough the parasite cell cycle by promoting arrest in S/M phase. To corroborate this evidence, we showed that the *Copaifera* oleoresin treatments were able to promote apparent ultra-structural parasite modifications. In general, we observed a possible compromising of endodyogeny, which was evidenced by the parasites bound by their basal ends. Moreover, oleoresin treatments promoted the formation of the multiple intracellular vacuole-like structures, as well as the presence of a single membrane in some areas, raising the possibility that the activity of these oleoresins can be related to some kind of DNA damage or replicative stress in the parasite. This hypothesis is corroborated by the results showing irreversible oleoresin inhibition of parasite proliferation. Besides, parasites from oleoresins-treated BeWo cells also had an impairment in their ability to infect new host cells, thus highlighting that the oleoresin treatment is probably causing some damage to parasites, which could be compromising the parasite physiology.

After evaluating the effects of *Copaifera* oleoresins against *T. gondii* infection in BeWo cells, we also studied the action of these same oleoresins using human villous explants from the third trimester of pregnancy. The concentration used for all oleoresins (128 µg/mL) was selected based on our results of LDH and MTT assays showing no significant toxicity to villous explants. As expected, we saw no morphological alterations of the villous exposed to oleoresins, at the set concentration. In order to corroborate with our in vitro findings, we demonstrated that the treatment with all four oleoresins (128 µg/mL) was able to inhibit *T. gondii* intracellular proliferation in villous explants. Interestingly, *C. pubiflora* oleoresin appeared to be more efficient in the inhibition of parasite growth than classical drugs (SDZ + PYR). As expected, SDZ + PYR treatment did not show cytotoxicity in villous explants and inhibited the intracellular proliferation of the parasites^33^.

Even though the mechanism of action of oleoresins appears to target the parasites, an additional and non-exclusive hypothesis to explain the impact of oleoresins against *T. gondii* could involve the modulation of the host cell environment. In order to support this hypothesis, we demonstrated that the oleoresins were capable of altering the cytokine production by both BeWo cells and villous explants infected or not by *T. gondii*. In general, we observed that the oleoresin treatments promoted a strong up-regulation of IL-6, regardless of the presence or absence of parasites in BeWo cells. Moreover, we showed that oleoresins (*C. duckei* and *C. pubiflora*) increase the MIF levels by uninfected cells. In agreement with the literature, *T. gondii* infection increased MIF production^33^. However, the treatment with oleoresins from *C. duckei* (32 µg/mL), *C. paupera* (64 and 32 µg/mL), and *C. pubiflora* (32 µg/mL) reduced MIF production by parasite-infected BeWo cells. Additionally, oleoresins treatments in specific concentrations down-modulated ROS production by uninfected and infected BeWo cells. Kian and colleagues corroborate these findings (2018)^53^ demonstrating that the treatment with *Copaifera martii* oleoresin in highest concentration reduced ROS production in *T. cruzi*-infected macrophages.

We reported that both uninfected and infected villous treated with all four oleoresins exhibited no significant alteration in the IL-6 and MIF production. However, TNF-α production was not detected in supernatants from the uninfected group, but the treatment with all four oleoresins was able to reduce the TNF-α release, primarily caused by *T. gondii* infection. In the context of the maternal–fetal interface, previous independent studies have reported that IL-6 and MIF are cytokines essentials to impair *T. gondii* infection. It was demonstrated by our research group that IL-6 is a multifaceted cytokine involved in the control of *T. gondii* infection in human monocytes and human trophoblastic cells^72,73^. Besides, this cytokine was also associated with the parasite control in infected-villous explants^30,31,33^. Several studies have demonstrated that human trophoblastic cells and human villous explants produce MIF and, this cytokine release has an important role during infection by different parasites, including *T. gondii.* Besides that, this cytokine has a critical function to reduce *T. gondii* proliferation in maternal–fetal interface^29,31,33,74–77^.

Thus, based on the literature and our data, we proposed that the modulation of cytokine production in BeWo cells during *T. gondii* infection by oleoresins is dependent on both the *Copaifera* species and the concentration of oleoresin used. Even though the IL-6 production appears to be a key cytokine related to parasite growth, our data suggest an alternative mechanism for the oleoresins to alter the host cell environment and therefore impair parasite growth. In this study, we demonstrated no significant differences in the production of IL-6 and MIF by uninfected and infected villous explants treated with oleoresins, but the oleoresin treatments significantly decreased TNF-α production in villous explants infected by *T. gondii*.

Taken together and despite the increase in IL-6 and MIF production by BeWo cells during parasite infection and under some oleoresin treatment conditions, in general, we observed that the oleoresin induced an anti-inflammatory profile to control parasite infection. This observation is in agreement with the literature, in which several publications have demonstrated that *Copaifera* oleoresins, including *C. cearensis*, *C. duckei*, *C. langsdorffii*, *C. multijuga*, *C. officinalis*, and *C. reticulata* have shown anti-inflammatory and antiparasitic properties^67^. In this line of discussion, we demonstrated that the treatment with both azithromycin and spiramycin (macrolide antibiotics) could control *T. gondii* infection in BeWo cells by inducing anti-inflammatory response with increased of MIF, IL-4, and IL-10 levels^29,78^. Acute *T. gondii* infection is marked by a high production of pro-inflammatory abortogenic cytokines, such as IFN-γ and TNF-α, which are deleterious for conception^79^. Thus, oleoresin treatment induces an anti-inflammatory response, which may be essential for the maintenance of gestation as well as to control the parasite infection.

In conclusion, the present study demonstrated that the oleoresins from *C. reticulata*, *C. duckei*, *C. paupera,* and *C. pubiflora* could control *T. gondii* infection as shown through both in vitro and ex vivo experimental models. We concluded that the anti-*T. gondii* effects triggered by these oleoresins are probably related to the immunomodulation of the host cells as well as the direct effect on parasites. Thus, our findings provide evidence of the potential for oleoresins to be an alternative source for the treatment for congenital toxoplasmosis by reducing the proliferation rate of *T. gondii* with low cytotoxicity effects to the host cells. Additional mechanisms by which the *Copaifera* oleoresins affect *T. gondii* need to be thoroughly evaluated in further studies.

## Methods

### Cell culture and parasite maintenance

Human trophoblast cells (BeWo lineage) were commercially purchased from the American Type Culture Collection (ATCC, Manassas, VA, USA) and maintained as previously described^32^. RPMI 1,640 medium (Cultilab, Campinas, SP, Brazil) supplemented with 100 U/mL penicillin (Sigma Chemical Co., St. Louis, MO, USA), 100 μg/mL streptomycin (Sigma) and 10% fetal bovine serum (FBS) (Cultilab) was used for cell culture maintenance. Cultures were kept at 37 °C under a humidified atmosphere containing CO_2_ (5%). *Toxoplasma gondii* tachyzoites (virulent RH strain, 2F1 clone) constitutively expressing the β-galactosidase gene were cultured as described elsewhere^73,80^. Briefly, tachyzoites were maintained by serial passages in BeWo cells cultured in RPMI 1,640 medium containing 2% FBS, 100 U/mL penicillin, and 100 μg/mL streptomycin at 37 °C and 5% CO_2_.

### *Copaifera* spp. oleoresins collection

The oleoresins used in the present study were collected between 2011 and 2014 in Northern region of Brazil, as following: *Copaifera reticulata* Ducke (Brasil Novo, Pará State, 03° 22.028′ S, 52° 29.947′ W), *Copaifera duckei* Dwyer (Belém, Pará State, 01° 06.933′ S, 48° 19.781′ W), *Copaifera paupera* Herzog (Xapuri, Acre State, 10° 27.861′ S, 68° 35.815′ W), and *Copaifera publifora* Benth (Mucajaí, Roraima State, 02° 36.205′ N, 60° 56.767′ W). The botanical identification was carried out by Silvane Tavares Rodrigues at the IAN Herbarium (EMBRAPA Amazônia Oriental), from who a taxonomic certification is available upon request, and the voucher specimens were deposited under the registry numbers 175266, 175206, 175983, and 180231, respectively for *C. reticulata*, *C. duckei*, *C. paupera*, and *C. pubiflora*. Brazilian Council for Authorization and Information on Biodiversity (SIBIO/ICMBio/MMA/BRASIL) and Genetic Heritage Management (CGEN/MMA/BRASIL) provided the legal permits for research activities with these plant materials in this paper. Permit numbers 35143-1 and 010225/2014-5, respectively.

### Host cell viability

We evaluated the host cell viability in the presence of the oleoresins to establish the non-toxic concentration of each oleoresin. The viability of BeWo cells treated with different concentrations of oleoresins was assessed by MTT colorimetric assay^81^. In summary, we tested four oleoresins from different species from *Copaifera* genus as follows: the oleoresins of *Copaifera reticulata*, *Copaifera duckei*, *Copaifera paupera,* and *Copaifera pubiflora* were solubilized in dimethyl sulfoxide (DMSO) and diluted in supplemented RPMI 1,640 medium to form a stock solution of 640 µg/mL^51,82^. BeWo cells were seeded at 3.0 × 10^4 ^cells/well in 96-well microplates. After adhesion, the culture monolayer was carefully rinsed, and solutions of oleoresins ranging from 256 to 4 µg/mL (twofold serial dilutions) were added to the microplate in the total volume of 200 μL/well. To assess whether DMSO toxicity in BeWo cells, we treated the cells with DMSO 1.2% in RPMI 1,640 medium in the total volume of 200 μL/well, which is the percentage of DMSO used in the highest oleoresin concentration (256 µg/mL). Culture medium alone was used as a positive control of viability, and cells considered to be 100% viable. We referred to published data to establish the work concentration of sulfadiazine (Sigma) and pyrimethamine (Sigma) (200 + 8 μg/mL, respectively), the chosen concentrations have been reported as non-toxic for BeWo cells^33^. Microplates were incubated for 24 h at 37 °C under a humidified atmosphere and 5% CO_2_. Next, we remove the supernatants and incubate the cells with MTT reagent (5 mg/mL, 10 µL) plus 90 µL of supplemented RPMI 1,640 medium for four hours at 37 °C and 5% CO_2_, followed by addition of 10% sodium dodecyl sulfate (SDS, Sigma) and 50% N,N-dimethyl formamide (Sigma) and further incubation for 30 min. MTT reduction was measured at 570 nm absorbance with a multi-well scanning spectrophotometer (Titertek Multiskan Plus, Flow Laboratories, McLean, VA, USA). We reported cell viability in percentages (cell viability %), with the absorbance of cells incubated only with culture medium considered to be 100% viable.

### *T. gondii* intracellular proliferation assay: β-galactosidase activity-based screen

We screened different oleoresin concentrations of *C. reticulata*, *C. duckei*, *C. paupera,* and *C. pubiflora* for their effect on growth modulation of a highly virulent strain (RH strain, 2F1 clone) of *T. gondii* using BeWo cells as host cell and a β-galactosidase colorimetric assay as previously described^72,73,77^. Briefly, BeWo cells were plated at 3.0 × 10^4 ^cells/well in 96-well microplates. After adhesion, cells were infected with *T. gondii* tachyzoites at a multiplicity of infection (MOI) of 3:1 (ratio of parasites per cell) in supplemented RPMI 1,640 medium. After three hours, the medium was discarded, and the cells rinsed with culture medium in order to remove the excess of extracellular parasites. Next, we incubated the cells for 24 h at 37 °C and 5% CO_2_ with non-toxic oleoresin concentrations in twofold serial dilutions as follows: *C. reticulata* and *C. paupera* (128 to 4 µg/mL), *C. duckei* and *C. pubiflora* (64 to 4 µg/mL). According to the literature, sulfadiazine, and pyrimethamine (SDZ + PYR) are considered classic drugs in the treatment of congenital toxoplasmosis. In this sense, the present work used this classic drug combination as gold-standard to evaluate the effect of the oleoresins^33^. Infected BeWo cells were incubated with RPMI 1,640 medium in the absence of any drug and used as negative treatment control. After 24 h of treatment, culture supernatants were collected and stored at − 80 °C for further measurement of cytokines. Next, *T. gondii* intracellular proliferation was analyzed using a colorimetric β-galactosidase assay. We quantified *T. gondii* intracellular proliferation in percentage (% of *T. gondii* proliferation) and calculated the number of tachyzoites in comparison to a standard curve produced with free tachyzoites (ranging from 1 × 10^6^ to 15.625 × 10^3^ parasites). Infected BeWo cells treated with only culture medium (negative control) represented non-inhibited parasite growth. We measured the efficiency of each treatment condition by comparison with the negative control (100% parasite proliferation).

### Adhesion assay of oleoresins-pretreated *T. gondii*

To investigate whether the effects of oleoresins occurs directly on the parasite, we performed an adhesion assay^83,84^ with minor modifications. BeWo cells were seeded at a density of 1.0 × 10^5^ cells in 24-well microplates containing 13 mm coverslips on each well. After adhesion, cells were fixed with paraformaldehyde (4%) for 30 min at room temperature and washed three times with 1 × phosphate-buffered saline (PBS). Next, *T. gondii* tachyzoites at an MOI of 3:1 were pre-incubated with oleoresins (64 and 32 µg/mL) from *C. reticulata*, *C. duckei*, *C. paupera,* and *C. pubiflora*; SDZ + PYR (200 + 8 μg/mL) or culture medium only (untreated group) for one hour at 37 °C and 5% CO_2_. We used the oleoresin concentrations stipulated on our preliminary results from the β-galactosidase assay. The pretreated parasites were rinsed and resuspended in the medium before incubation with cells for three hours at 37 °C and 5% CO_2_. After removing the excess of *T. gondii* that did not adhere to cells with repeated rinsing with 1 × PBS, parasites were fixed in the same conditions as mentioned above. The coverslips were then incubated overnight with a mouse monoclonal primary anti-*Toxoplasma gondii* antibody [SAG1/p30] (Abcam TP3 #ab8313) (diluted 1: 500 in PGN-0.01% saponin solution). Finally, coverslips were rinsed three times with 1 × PBS, and incubated with Alexa Fluor 488-conjugated anti-mouse IgG (Invitrogen, USA #A11001) (diluted 1:500 in PGN-0.01% saponin solution), tetramethylrhodamine isothiocyanate (TRITC)-conjugated phalloidin (Sigma, P1951) (diluted 1:50 in PGN + saponin) and 4,6′-diamidino-2-phenylindole dilactate (DAPI, Invitrogen, USA) (diluted 1:500 in PGN + saponin) for one h in the dark at room temperature to label tachyzoites of *T. gondii*, F-actin and nuclei, respectively. We mounted the Coverslips onto glass slides, and samples were analyzed using confocal fluorescence microscopy (Zeiss, LSM 510 Meta, Germany) with an inverted microscope (Zeiss Axiovert 200 M). We analyzed the following parameters: the number of BeWo cells with adhered parasites and the total number of attached parasites per cell in a total of 20 fields chosen randomly.

### Invasion and intracellular proliferation of oleoresins-pretreated *T. gondii*

After verifying the adhesion of pretreated parasites in BeWo cells, we also investigated the invasion and intracellular proliferation of oleoresins-pretreated parasites. The experimental procedures to assess the possible direct effect of oleoresins on *T. gondii* tachyzoites were performed according to Barbosa et al*.* (2012)^32^ and Adeyemi et al*.* (2017)^36^, with minor modifications. In the first set of experiments, BeWo cells were seeded at 3.0 × 10^4 ^cells/well in 96-well microplates for 24 h in supplemented RPMI 1,640 medium. Next, cell culture-derived tachyzoites of *T. gondii* at a ratio of 3 parasites per cell (3:1) were pre-incubated for one hour at 37 °C and 5% CO_2_ with two oleoresin concentrations (64 and 32 µg/mL) from *C. reticulata*, *C. duckei*, *C. paupera,* and *C. pubiflora*. At the same conditions, parasites were also pre-incubated with SDZ + PYR (200 + 8 μg/mL) or with only culture medium (untreated group). Afterward, purified parasite suspension in a treatment-free medium was placed in 96-well tissue culture plates containing previously adhered BeWo cells, and invasion was allowed to occur for three hours. Following this basic design setup, we tested two experimental conditions; (1) for the invasion analysis, after 3 h of incubation, the monolayers were thoroughly rinsed in order to remove the extracellular parasites; (2) for intracellular proliferation analysis, after 3 h of incubation, the monolayers were rinsed, and a fresh supplemented culture medium was added for an additional 24 h at 37 °C and 5% CO_2_. In both experiments, intracellular *T. gondii* was quantified using the β-galactosidase assay.

### Reversibility assay

In order to evaluate the maintenance of the antiparasitic effects of oleoresins on *T. gondii* proliferation, we performed the reversibility assay as previously described^36,85^, with minor modifications. Briefly, BeWo cells were seeded at 3.0 × 10^4 ^cells/well in 96-well microplates. After adhesion, cells were infected with *T. gondii* tachyzoites at an MOI of 3:1 (parasites: cell) in supplemented RPMI 1,640 medium. After three hours of invasion, we rinsed the cells to remove the unattached parasites and used this basic experimental setup to test two conditions, as follows; (1) the invading parasites (after three h of invasion) were allowed to grow in the presence of oleoresins (64 and 32 µg/mL) from *C. reticulata*, *C. duckei*, *C. paupera,* and *C. pubiflora*, SDZ + PYR (200 + 8 μg/mL) or culture medium only (untreated group) for 24 h at 37 °C and 5% CO_2_. In the second condition tested, (2) the invading parasites were grown at the same conditions of treatments (as mentioned above on item 1), however after 24 h of treatment, the cells were rinsed, the media replaced by complete RPMI 1,640, and the parasites allowed to growth for an additional 24 h. In both situations, we quantified *T. gondii* intracellular proliferation using the β-galactosidase assay, as mentioned above. Finally, we measure the reversibility rate in percentage (reversibility of treatment %) in 24 h after treatment removal by comparison with both the untreated group (considered as 100% of reversibility) and the corresponding treatment condition in 24 h of treatment (baseline for comparison).

To corroborate the reversibility data, we investigated whether the oleoresin treatment of infected BeWo cells with *T. gondii* tachyzoites would interfere in the ability of these parasites to invade and replicate inside new fresh cells. In summary, BeWo cells were seeded at 1.0 × 10^6 ^cells/well in 6-well microplates. After adhesion, cells were infected with *T. gondii* tachyzoites at an MOI of 3:1 (parasites: cell) in supplemented RPMI 1,640 medium for three h at 37 °C and 5% CO_2_. Next, we treated the infected cells with oleoresins (64 and 32 µg/mL) from *C. reticulata*, *C. duckei*, *C. paupera,* and *C. pubiflora*; SDZ + PYR (200 + 8 μg/mL) or culture medium only (untreated group) for 24 h at 37 °C and 5% CO_2_. After, we isolated the intracellular parasites from infected cells by multiple passages through a 21 and 26-gauge needle. Finally, *T. gondii* tachyzoites from each experimental condition were then allowed to reinfect monolayers of BeWo cells previously seeded in 96-well microplates (3.0 × 10^4 ^cells/well). We used the β-galactosidase assay to quantify the tachyzoites after 24 h incubation.

### Parasite cell cycle analysis

The parasite cell cycle was analyzed by flow cytometry as previously described^69,70,86^, with some modifications. Briefly, BeWo cells were seeded at 1.0 × 10^6 ^cells/well in 6-well microplates. After adhesion, cells were infected with *T. gondii* tachyzoites at an MOI of 3:1 (parasites: cell) in supplemented RPMI 1,640 medium for three h at 37 °C and 5% CO_2_. Next, we treated the cells with oleoresins (64 and 32 µg/mL) from *C. reticulata*, *C. duckei*, *C. paupera,* and *C. pubiflora*; SDZ + PYR (200 + 8 μg/mL) or culture medium only (untreated group) for 24 h at 37 °C and 5% CO_2_. After, we isolated the intracellular parasites from infected cells by multiple passages through a 21 and 26-gauge needle. *T. gondii* tachyzoites from each experimental condition were rinsed with PBS and then fixed overnight with ethanol (70%) at 4 °C. Next, fixed parasites were rinsed and incubated overnight with a mouse monoclonal primary anti-*Toxoplasma gondii* antibody [SAG1/p30] (Abcam TP3 #ab8313) (diluted in 1 × PBS). Finally, parasites were rinsed with 1 × PBS, incubated with Alexa Fluor 488-conjugated anti-mouse IgG (Invitrogen, USA #A11001), 100 mg/mL of RNase A and 10 µg/mL of PI, all reagents diluted in 1 × PBS. We labeled the parasites in the dark for 45 min at room temperature. Quantification of the DNA content for parasite cycle analysis was performed using the Cytoflex flow cytometer (Beckman Coulter, United States), and the data collected by using FlowJo software (version 7.6.3).

### Transmission electron microscopy (TEM)

Transmission electron microscopy (TEM) studies were carried out as previously described^33,87^, with the following minor changes. Briefly, BeWo cells were seeded at 1.0 × 10^6 ^cells/well in 6-well microplates. After adhesion, cells were infected with *T. gondii* tachyzoites at an MOI of 3:1 (parasites: cell) in supplemented RPMI 1,640 medium. After three h of invasion, cells were rinsed and incubated for 24 h at 37 °C and 5% CO_2_ with oleoresins (64 µg/mL) from *C. reticulata*, *C. duckei*, *C. paupera,* and *C. pubiflora*; SDZ + PYR (200 + 8 μg/mL) or culture medium only (untreated group). Next, we harvested the cells and fixed them in Karnovsky solution containing 2% paraformaldehyde and glutaraldehyde in a 0.1 M sodium cacodylate buffer (pH 7.4) for 24 h. Samples were incubated for one h in 1% osmium tetroxide (OsO_4_) in cacodylate solution and treated with potassium ferrocyanide for an additional 30 min at room temperature., followed by dehydration in increasing concentrations of ethanol and propylene oxide, and inclusion in Epon resin. Ultrathin sections were stained with uranyl acetate and lead citrate and then analyzed using a transmission electron microscope (Hitachi, TM 3000).

### Measurement of intracellular reactive oxygen species (ROS) by BeWo cells

The influence of oleoresins from different species of *Copaifera* in ROS production by BeWo cells was based on the intracellular peroxide-dependent oxidation of 2′,7′-dichlorodihydrofluorescein diacetate (H_2_DCF-DA) (Invitrogen|catalog number: D399) to form the fluorescent compound 2′,7′-dichlorofluorescein (DCF), as previously described^53,88^, with some modifications. Briefly, BeWo cells were seeded at 0.7 × 10^5 ^cells/well in 24-well microplates. Then, cells were infected or not with *T. gondii* tachyzoites in a ratio of 3:1 (parasites: cell) for 3 h in a supplemented RPMI 1,640 medium at 37 °C and 5% CO_2_. Subsequently, cells were rinsed thoroughly with culture medium and treated with oleoresins (64 and 32 µg/mL) from *C. reticulata*, *C. duckei*, *C. paupera,* and *C. pubiflora*; SDZ + PYR (200 + 8 μg/mL) or culture medium only (untreated group) for 24 h at 37 °C and 5% CO_2_. Hydrogen peroxide (H_2_O_2_) was used as a positive control of ROS production. After treatment, cells were harvested, rinsed with 1 × PBS, and incubated with 150 μL of H_2_DCF-DA (10 μM; diluted in 1 × PBS containing 10% FBS) for 45 min at 37 °C and 5% CO_2_ under darkness. Finally, DCF fluorescence intensity was detected immediately using a Cytoflex flow cytometer (Beckman Coulter, United States) and FlowJo software (version 7.6.3). Data are presented as median fluorescence intensity (MFI).

### Human chorionic villous explant culture

In order to extrapolate our in vitro findings obtained in human trophoblast cells, we assessed the effects of oleoresins from different species of *Copaifera* during *T. gondii* infection using an ex vivo model. We used human villous explants from the placentas in the third trimester, as this model is a good representation of the maternal–fetal interface^30,76^. Firstly, third-trimester human placentas (36 to 40 weeks of pregnancy) were collected after elective cesarean section deliveries at the Clinics Hospital of the Federal University of Uberlândia (HC-UFU), MG, Brazil. Placental tissues were collected following exclusion criteria, as follows: pre-eclampsia, chronic hypertension, infectious disease including toxoplasmosis, chorioamnionitis, chronic renal disease, cardiac disease, connective tissue disease, pre-existing diabetes mellitus, gestational diabetes mellitus, and other pathological manifestations. In brief, placental tissues were washed in ice-cold sterile PBS (pH = 7.2) in order to remove excess blood, and then aseptically dissected to remove endometrial tissue and fetal membranes up to 1 h after collection. Terminal chorionic villi containing five to seven free tips per explant were collected as described previously^89^ and added to 96-well microplates (one villus per well) in 200 µL/well of a fresh RPMI 1,640 medium supplemented with 100 U/mL penicillin, 100 μg/mL streptomycin (Sigma) and 10% FBS for 24 h at 37 °C under a humidified atmosphere containing CO_2_ (5%) until the experimental procedure.

### Viability of human chorionic villous explant treated with oleoresins

Oleoresin toxicity in human villous explants was performed using viability assays with both a lactate dehydrogenase (LDH) and MTT assays, according to published protocols^30,81,90^. In both assays, villous explants were collected and cultured in a supplemented RMPI 1,640 medium for 24 h at 37 °C and 5% CO_2,_ as mentioned above. After 24 h, villi were treated oleoresin of *C. reticulata*, *C. duckei*, *C. paupera,* and *C. pubiflora* at 256, 128, and 64 µg/mL or SDZ + PYR (150 and 200 µg/mL, respectively). These drug concentrations were chosen based on the previous study by our research group, showing that the drugs at the mentioned concentration have no significant toxicity in human villous explants^33^. As vehicle control, we treated the villi with DMSO (1.2%) in RPMI 1,640 medium, which is the percentage of DMSO used in the highest oleoresin concentration (256 µg/mL). As control of viability, villous explants were treated with culture medium alone. Following 24 h of incubation, the culture supernatants were collected, and LDH concentration measured according to the manufacturer’s instructions^30^ with minor modifications (LDH Liquiform, Labtest Diagnostica S.A., Lagoa Santa, MG, Brazil|Ref 86-2/30, Lot 7010). This assay is based on the consumption and decrease of absorption of NADH at 340 nm, as measured by a microplate reader (Versa Max ELISA Microplate Reader, Molecular Devices, Sunnyvale, CA, USA). LDH released into the culture media was expressed in U/L of LDH enzymatic activity and was used as a marker for tissue integrity.

In parallel, at the same experimental condition, as described above, tissue viability was also assessed by the MTT assay. After 24 h of treatment, culture supernatants were discarded and replaced by 200 µL MTT solution [180 µL culture medium plus 20 µL MTT (5 mg/mL)] for 4 h at 37 °C and 5% CO_2_. Subsequently, formazan crystal resulting from MTT reduction was solubilized by the addition of 100 µL PBS containing sodium dodecyl sulfate (SDS, 10%) and HCl (0.01 M, 18 h, 37 °C, 5% CO_2_). Finally, villi were removed from each well, and the absorbance (570 nm) was measured with a multi-well scanning spectrophotometer (Titertek Multiskan Plus, Flow Laboratories, McLean, VA, USA). Tissue viability was expressed in percentages (viability % by MTT incorporation), with the absorbance of villi incubated with culture medium alone (untreated group) considered to be 100% viable. Furthermore, we performed morphological analysis of treated villi to corroborate the viability assays. Villi tissue sections were stained with hematoxylin/eosin and examined using a light microscope (BX40 Olympus, Tokyo, Japan)^30^.

### *T. gondii* infection of human chorionic villous explants and oleoresin treatments

We quantified *T. gondii* intracellular proliferation in human villous explants treated with oleoresins using a colorimetric β-galactosidase assay^30,76^, with minor modifications. In brief, villi were collected and cultured in 96-well microplates (one villus per well/200 µL) in a supplemented culture medium for 24 h at 37 °C and 5% CO_2_. Next, the villi were infected with *T. gondii* tachyzoites of RH strain-2F1 (1 × 10^6^ parasites per well/200 µL) and incubated for 24 h at 37 °C and 5% CO_2_. Afterward, villous explants were rinsed thoroughly with culture medium in order to remove non-adhered parasites. Following our data from tissue viability (LDH and MTT assays), the villi were treated for an additional 24 h with oleoresins (128 µg/mL) from *C. reticulata*, *C. duckei*, *C. paupera* and *C. pubiflora*, and SDZ + PYR (150 + 200 μg/mL). At the same experimental conditions, uninfected and untreated villous explants or infected and untreated villous explants were cultured with culture medium alone. Finally, culture supernatants were collected and stored at − 80 °C for cytokines measurement. In addition, villous explants were also collected and stored at − 80 °C for the following analyzes: protein content determination using Bradford reagent and *T. gondii* intracellular proliferation by β-galactosidase assay.

Initially, frozen villous explants were homogenized by addition of 150 µL of radioimmunoprecipitation assay buffer (RIPA) [50 mM Tris-HCl, 150 mM NaCl, 1% Triton X-100, 1% (w/v) sodium deoxycholate, and 0.1% (w/v) SDS, pH 7.5] containing protease inhibitor cocktail (Complete, Roche Diagnostic, Mannheim, Germany). The homogenate was centrifuged at 21,000× *g* for 15 min at 4 °C, and the supernatant was collected to measure the total amount of protein using the Bradford method^91^. Determination of *T. gondii* intracellular proliferation in villous explants was carried out with 20 µL of supernatants from each sample. The supernatants were incubated with 160 μL of assay buffer (100 mM 1 × PBS, pH 7.3, 102 mM β-mercaptoethanol and 9 mM MgCl_2_) and 40 μL of 6.25 mM CPRG (chlorophenol red-β-D-galactopyranoside; Roche, Indianapolis, IN) for approximately 30 min. We measured the absorbance at 570 nm using a kinetic plate reader (Titertek Multiskan Plus, Flow Laboratories, McLean, VA, USA). The number of *T. gondii* tachyzoites were normalized according to the total protein concentration (µg/mL) of each villous obtained by Bradford assay, and expressed by the number of parasites per µg of tissue. *Toxoplasma gondii* intracellular proliferation in villous explants was expressed in percentage (% of *T. gondii* proliferation), and the number of tachyzoites calculated in comparison to a standard curve of free tachyzoites (ranging from 1 × 10^6^ to 15.625 × 10^3^ parasites). As controls we quantified the number of tachyzoites from untreated and infected villous incubated with culture medium alone (negative control), this condition was considered as 100% of parasite proliferation, and the number of parasites from each treatment condition was expressed in percentage of *T. gondii* proliferation in comparison with the negative control.

### Cytokine determination

We measured the release of cytokines in supernatants of BeWo cells and villous explants cultures using a double-antibody sandwich enzyme-linked immunosorbent assay (ELISA). Assays for IL-6, TNF-α, IL-10, TGF-β1 (OpTEIA, BD Bioscience, San Diego, CA, USA) and MIF (Duoset R&D Systems, Minneapolis, MN, USA) were performed according to the manufacturer’s instructions. Cytokine concentrations for experiments with BeWo cells were expressed in pg/mL. In contrast, we normalized the concentration fo cytokines in the villous experiments using a ratio between cytokine production (pg/mL) and its corresponding total protein content (mg/mL) of each villous, as obtained by the Bradford method for protein quantification and results are shown as pg/mg of tissue. The limits of detection of each cytokine were determined from standard curves: IL-6 (4.7 pg/mL), TNF-α (7.8 pg/mL), MIF (7.8 pg/mL), IL-10 (7.8 pg/mL) and TGF-β1 (125 pg/mL).

### Statistical analysis

All data were expressed as means ± standard deviations (SD). Data were checked first for normal distribution. Significance differences were compared to controls by using One-way ANOVA, Tukey’s, or Sidak’s multiple comparisons post-test for the parametric data. Nonparametric data were analyzed by the Kruskal–Wallis test and Dunn’s multiple comparison post-test. Data were considered statistically significant at *P* < 0.05, according to the experimental design (GraphPad Prism Software version 6.01).

### Ethical approval

The present research protocol using human tissue samples was performed in accordance with relevant guidelines and regulations, and the experimental protocols were approved by the Ethics Committee of the Federal University of Uberlandia, MG, Brazil, with Approval Number 1.585.342. A consent term was obtained from all subjects, and when the subjects were under 18, a parent and/or legal guardian was acquired. In addition, informed consent was obtained from all participants and/or their legal guardians.
